# Osteocyte Estrogen Receptor β (Ot‐ERβ) Regulates Bone Turnover and Skeletal Adaptive Response to Mechanical Loading Differently in Male and Female Growing and Adult Mice

**DOI:** 10.1002/jbmr.4731

**Published:** 2022-12-19

**Authors:** Xiaoyu Xu, Haisheng Yang, Whitney A. Bullock, Maxim A. Gallant, Claes Ohlsson, Teresita M. Bellido, Russell P. Main

**Affiliations:** ^1^ Weldon School of Biomedical Engineering Purdue University West Lafayette IN USA; ^2^ Musculoskeletal Biology and Mechanics Lab, Department of Basic Medical Sciences Purdue University West Lafayette IN USA; ^3^ Department of Biomedical Engineering, Faculty of Environment and Life Beijing University of Technology Beijing China; ^4^ School of Medicine, Indiana University Bloomington IN USA; ^5^ Sahlgrenska Osteoporosis Centre, Centre for Bone and Arthritis Research, Department of Internal Medicine and Clinical Nutrition Institute of Medicine, Sahlgrenska Academy, University of Gothenburg Gothenburg Sweden; ^6^ Department of Drug Treatment Sahlgrenska University Hospital Gothenburg Sweden; ^7^ Department of Physiology and Cell Biology University of Arkansas for Medical Sciences Little Rock AR USA

**Keywords:** ESTROGEN RECEPTOR, OSTEOCYTES, BONE TURNOVER, SKELETAL ADAPTATION, MECHANICAL LOADING

## Abstract

Age‐related bone loss is a failure of balanced bone turnover and diminished skeletal mechanoadaptation. Estrogen receptors, ERα and ERβ, play critical roles in osteoprotective regulation activated by estrogen and mechanical signals. Previous studies mainly focused on ERα and showed that osteocyte‐ERα (Ot‐ERα) regulated trabecular, but not cortical bone, and played a minor role in load‐induced cortical adaptation. However, the role of Ot‐ERβ in bone mass regulation remains unrevealed. To address this issue, we characterized bone (re)modeling and gene expression in male and female mice with Ot‐ERβ deletion (ERβ‐dOT) and littermate control (LC) at 10 weeks (young) or 28 weeks (adult) of age, as well as their responses to *in vivo* tibial compressive loading. Increased cancellous bone mass appeared in the L_4_ vertebral body of young male ERβ‐dOT mice. At the same time, femoral cortical bone gene expression showed signs consistent with elevated osteoblast and osteoclast activities (type‐I collagen, Cat K, RANKL). Upregulated androgen receptor (AR) expression was observed in young male ERβ‐dOT mice relative to LC, suggesting a compensatory effect of testosterone on male bone protection. In contrast, bone mass in L_4_ decreased in adult male ERβ‐dOT mice, attributed to potentially increased bone resorption activity (Cat K) with no change in bone formation. There was no effect of ERβ‐dOT on bone mass or gene expression in female mice. Sex‐dependent regulation of Ot‐ERβ also appeared in load‐induced cortical responsiveness. Young female ERβ‐dOT mice showed an enhanced tibial cortical anabolic adaptation compared with LC. In contrast, an attenuated cortical anabolic response presented at the proximal tibia in male ERβ‐dOT mice at both ages. For the first time, our findings suggest that Ot‐ERβ regulates bone (re)modeling and the response to mechanical signals through different mechanisms in males and females. © 2022 The Authors. *Journal of Bone and Mineral Research* published by Wiley Periodicals LLC on behalf of American Society for Bone and Mineral Research (ASBMR).

## Introduction

Age‐related bone loss in males and females is recognized as a lack of osteoprotective metabolisms by declining sex hormones, mainly estrogen,^(^ ^1^, ^2^ ^)^ and failure of the skeletal adaptative response to mechanical stimuli.^(^ ^3^, ^4^, ^5^ ^)^ Severe bone loss leads to osteoporosis and causes more than 2 million skeletal fractures in the United States every year in people older than 50 years.^(^ ^6^ ^)^ Osteocytes (Ot) are critical regulators of bone metabolism through orchestrating osteoblast and osteoclast differentiation and activities in response to biochemical and mechanical signals.^(^ ^7^, ^8^ ^)^ Although estrogen and estrogen receptors (ERs) play a significant role in bone mass protection,^(^ ^9^, ^10^ ^)^ previous studies have discussed the minor effect of estrogen on skeletal adaptation, suggesting that osteocytes might sense and transduce mechanical signals by ERs through an estrogen‐independent mechanism.^(^ ^11^, ^12^, ^13^ ^)^

There are two subtypes of ERs (ERα and ERβ) in human and rodent bone tissues expressed by all bone cell types.^(^ ^14^, ^15^ ^)^ Both ERs are required for estrogen‐mediated osteoprotective regulation,^(^ ^16^, ^17^ ^)^ as well as osteocyte mechanosensing and skeletal mechanoadaptation.^(^ ^18^, ^19^, ^20^ ^)^ Global ER knockout (KO) mice showed the potentially opposing effects of ERα and ERβ on bone metabolism during growth, where ERα appears to protect cortical and trabecular bone in male and female mice,^(^ ^21^, ^22^ ^)^ whereas ERβ inhibits endocortical bone formation in female mice and does not affect trabecular bone mass in male or female mice.^(^ ^23^, ^24^ ^)^ Moreover, ERβ was indicated in global KO studies to diminish load‐induced cortical adaptation in female mice^(^ ^11^, ^25^ ^)^ and appears to suppress the osteogenic functions of ERα in response to mechanical signals.^(^ ^13^, ^18^, ^26^ ^)^ However, systemic hormonal changes (IGF‐1 and estrogen) and increased fat mass in global KO mice confound the interpretation of the local effects of ERα and ERβ on bone tissue.^(^ ^21^, ^25^, ^26^, ^27^, ^28^ ^)^ Therefore, conditional KO models of ERs in bone cells are needed to address the cell‐autonomous functions of ERs in mediating bone metabolism in response to estrogen or mechanical signals without causing systemic hormonal effects.^(^ ^28^, ^29^, ^30^ ^)^ Osteocyte‐ERα (Ot‐ERα) is shown to mediate cancellous, but not cortical bone morphology, for male and female mice during normal growth,^(^ ^31^, ^32^ ^)^ whereas the deletion of Ot‐ERα has no impact on the cortical mechanoadaptation in female mice.^(^ ^32^ ^)^ These minor roles of Ot‐ERα in cortical bone regulation suggest that Ot‐ERβ may dominate in mediating the cortical bone morphology and mechanical adaptation. Moreover, ERβ was proposed in previous global KO studies to mediate skeletal mechanoadaptation by sex and bone compartment^(^ ^11^, ^20^, ^25^ ^)^ and was shown *in vitro* to suppress strain‐induced osteoblast proliferation.^(^ ^20^ ^)^ However, specific *in vivo* studies revealing the function of Ot‐ERβ in bone growth and functional adaptation are lacking. Understanding the underlying mechanisms of ER signaling in bone metabolism and load‐induced skeletal osteogenic adaptation is fundamental for elucidating the role of sex hormones and their receptors in balancing growth and functional adaptation in male and female skeletons.

Our study aims to determine the role of ERβ in osteocytes on bone morphology and the anabolic response of the skeleton to mechanical loading in male and female mice. Given the results of previous skeletal *in vivo* ER studies, we hypothesize that ERβ in osteocytes mediates cortical, but not cancellous, bone morphology by sex and might exert an inhibitory regulation in load‐induced anabolic adaptation. We tested our hypotheses in male and female mice with Ot‐ERβ deletion generated using the *Dmp1*‐Cre system.^(^ ^28^, ^33^ ^)^ Mice were subjected to axial tibial compressive loading at 10 weeks or 28 weeks of age. We focused on relating the genetic and loading effects to the skeletal phenotype using micro‐computed tomography (micro‐CT) and detecting underlying cellular mechanisms through gene expression analysis.

## Materials and Methods

### Animal model generation

Osteocyte‐ERβ knockout mice (ERβ‐dOT) were generated by breeding mice with a floxed ERβ gene (*Esr2*) to *Dmp1*‐8 kb‐Cre mice. The use of the floxed ERβ mice was permitted by their creator, Dr Pierre Chambon,^(^ ^28^ ^)^ and obtained from Dr Sundeep Khosla (Mayo Clinic, Rochester, MN, USA). The offspring of this cross were bred to generate male and female ERβ‐dOT mice (KO, ERβ^Cre/+; fl/fl^) and littermate controls (LC, ERβ^−/−; fl/fl^). Using the *Dmp1*‐8 kb‐Cre model system, we generated mice with ERβ deletion in osteocytes,^(^ ^28^, ^33^, ^34^ ^)^ whereby the expression of *DMP1* promotes the Cre‐induced ERβ exon 3 deletions in the skeleton. Although *DMP1* is also expressed in late‐stage osteoblasts in the mammalian skeleton, previous studies have implicated that mature osteoblasts that produce *DMP1* are destined to differentiate into osteocytes.^(^ ^35^ ^)^ Primers for detecting the floxed ERβ were 5′‐GCATAGCGCAGTTGGTAGAG‐3′ (ERβ exon 3), 5′‐CTTCTTAGAGGTACGGATCCCAGCCC‐3′ (ERβ exon 3), and for the Cre transgene 5′‐CATCGCTCGACCAGTTTAGTTACC‐3′ (Cre), and 5′‐CATACCTGGAAAATGCTTCTGTCC‐3′ (Cre). Mice were housed 3 to 5 per cage with a 12‐hour light/dark cycle and *ad libitum* access to food (Teklad Rodent Diets, Envigo, Madison, WI, USA) and water. Newborn mice were genotyped and assigned to Ot‐ERβ and LC groups at 3 to 4 weeks of age. Mice were then aged to 10 weeks or 28 weeks of age. At no point in time were the primary investigators ignorant of the identity of the mice, as sample sizes were based on the accumulation of the correct genotypes for stiffness and finite element analysis (FEA), phenotyping, mechanical loading, gene expression, and blood serum analyses. The FEA was conducted on *n* = 3 mice per group. Analyses for phenotyping and mechanical loading were conducted on the same 10 to 12 mice per group. Gene expression analyses were conducted on 4 to 10 mice per group. Investigators conducting the blood serum analyses required *n* = 7–10 mice from the phenotyping/mechanical loading mice, which were selected randomly and analyzed in a blinded manner. All experimental procedures were approved by Purdue University's Animal Care and Use Committee (IACUC #1203000623).

### Strain gauge calibration for *in vivo* compressive loading experiments in ERβ‐dOT and LC mice

To determine the magnitude of the applied compressive loads to induce a tibial osteogenic response, *in vivo* load‐strain calibrations were conducted by applying a single element strain gauge to the medial surface of the tibial midshaft in male and female ERβ‐dOT and LC mice at 10 weeks and 28 weeks of age (*n* = 3 mice/group) following previous methods.^(^ ^36^, ^37^ ^)^ Data from the left and right tibias were averaged for each mouse before group means were calculated. The gauge‐measured stiffness was not different between genotypes in male and female mice at either age (Supplemental Table S1). Knowing that +1200 με applied at the medial tibial midshaft induces an osteogenic loading response,^(^ ^38^ ^)^ we targeted a higher strain (+1500 με) for the 10‐week‐old mice to ensure a response in both ERβ‐dOT and LC mice.^(^ ^39^ ^)^ Previous mouse studies have shown that the skeletons of older mice are less responsive to strain levels that are osteogenic in younger mice.^(^ ^3^, ^4^, ^39^ ^)^ Thus, greater medial diaphyseal strain levels (+1800 με) were targeted for the 28‐week‐old group. The choice of loads was based solely upon experimental diaphyseal strains, without strict requirements for genotype‐matched cancellous bone strains.^(^ ^40^ ^)^ Based upon the strain gauge‐based stiffness measures, the target strains would be induced at compressive loads of −9 N and −13 N for the 10‐week‐old female and male mice, respectively, and −11 N and −12.5 N for the 28‐week‐old female and male mice, respectively.

### Micro‐CT‐based finite element (FE) analysis

Left and right tibias with the strain‐gauge foils attached were scanned by micro‐CT with an isotropic voxel resolution of 10 μm (μCT40, Scanco Medical AG, Brüttisellen, Switzerland; 55 kVp, 145 mA, 300 ms integration time, no frame averaging). FE analysis was performed using micro‐CT scans to characterize the full‐field strain environments throughout the tibias following methods outlined in our previous studies (Abaqus 6.13.3, Simulia, Dassault Systemes, Dearborn, MI, USA).^(^ ^37^ ^)^ The FE‐predicted strain at the gauge site was validated by averaging the nodal strains aligned with the longitudinal axis of the overlying strain gauge. For stiffness and strain computed at the gauge site, there was no difference between the magnitudes given by FEA and the experimental measurements for each genotype‐sex combination in either age group (Supplemental Tables S1 and S2). Also, strain environments for the diaphyseal cortical and proximal cancellous bone of the gauged tibias were analyzed and compared across genotype and sex for each age group. Volumes of interest (VOIs) for the diaphyseal cortical bone spanned 2.5% of the average bone length and were centered at the mid‐diaphysis (50%) and a distance of 37% of the tibia's length from the proximal end.^(^ ^40^ ^)^ The VOI for the proximal metaphyseal cancellous bone began distal to the growth plate, excluding the primary spongiosa and cortical shell, and extended a total of 5% of the average bone length.^(^ ^40^, ^41^ ^)^ Peak principal strains were found using methods consistent with our previous studies and were defined using the cut‐off values for the upper 95th percentile of the maximum (tensile) or minimum (compressive) principal strains for each VOI.^(^ ^37^, ^40^ ^)^ At the beginning of the loading studies, the FE‐predicted strains in the cortical and cancellous VOIs did not differ by genotype (Supplemental Table S3).

### *In vivo* mechanical loading in ERβ‐dOT and LC mice

Male and female ERβ‐dOT and LC mice at 10 weeks and 28 weeks of age (*n* = 10–12/sex/genotype) were selected to represent young and adult mice, respectively. *In vivo* cyclic compressive loading was applied for 10 days over 2 weeks (Monday–Friday) to the left tibia (loaded) for each mouse (TestBench). On each day of loading, mice were anesthetized (2% isoflurane, 1.0 L O_2_/min) and a −1 N pre‐load was applied before applying 216 continuous compressive triangle‐waveform cycles per day with a 5‐second rest at −1 N inserted between every 4 load cycles (following protocol III).^(^ ^40^ ^)^ The right tibia served as a contralateral control. The actual loads achieved for the male and female mice were −8.9 N and −12.9 N for the 10‐week‐old female and male mice, respectively, and −11 N and −12.5 N for the 28‐week‐old female and male mice, respectively. After 2 weeks of loading, mice were anesthetized (4% isoflurane, 1.0 L O_2_/min) and euthanized at 12 weeks and 30 weeks of age through cardiac exsanguination. Left and right tibias and lumbar spines were dissected free of soft tissue, fixed in 10% neutral buffered formalin for 48 hours, and then stored in 70% ethanol at room temperature.

### Micro‐CT imaging and analyses

The loaded and control tibias of 12‐week‐old and 30‐week‐old ERβ‐dOT and LC mice were scanned by micro‐CT with the same settings as previously described in the FE analysis section. The cortical and cancellous VOIs presented for the bone morphology analyses correspond to the VOIs used in the FEA. Threshold values used to segment the cortical and cancellous compartments from soft tissues were 357 mg HA/cm^3^ and 224 mg HA/cm^3^, respectively, irrespective of age, sex, or genotype, and visualized by eye.^(^ ^43^ ^)^ The right (control) tibia was used to compare the effect of genotype (ERβ‐dOT versus LC) at 12 weeks and 30 weeks of age. This comparison assumes no global effect of unilateral tibial loading on the contralateral control limb.^(^ ^44^ ^)^ Lumbar spines were scanned by micro‐CT (Quantum GX, PerkinElmer, Waltham, MA, USA; 90 kVP, 88 μA, 25 mm FOV, high‐resolution sub‐volume reconstruction) for vertebral cortical and cancellous bone analyses. The fourth lumbar vertebra (L_4_) was reconstructed with an isotropic voxel resolution of 10 μm, and the vertebral body was analyzed using an adaptive thresholding algorithm in the manufacturer's software (AccuCT 1.1 beta, PerkinElmer).

Measured parameters for cortical bone analysis included cortical bone area (Ct.Ar; mm^2^), cortical bone volume (Ct.BV; mm^3^), cortical thickness (for L_4_ only; Ct.Th; mm), maximum and minimum moments of inertia (for tibias only; Imax, Imin; mm^4^), which are commonly used for representing the maximum and minimum directions for cross‐sectional structural rigidity in cortical bone, and cortical bone tissue mineral density (for tibias only, Ct.BMD, mg HA/mm^3^). Measurements for cancellous bone analysis included bone volume fraction (BV/TV; %), trabecular number (Tb.N; 1/mm), trabecular thickness (Tb.Th; mm), trabecular separation (Tb.Sp; mm or μm), trabecular bone volume (Tb.BV, mm^3^), trabecular tissue volume (Tb.TV, mm^3^), trabecular bone surface (for L_4_ only; Tb.BS, mm^2^), and trabecular bone tissue mineral density (Tb.BMD, mg HA/mm^3^).^(^ ^45^ ^)^

### Total RNA isolation and gene expression analyses

Femora and lumbar spines with surrounding musculature removed were collected from male and female ERβ‐dOT and LC mice at 12 weeks and 30 weeks of age (*n* = 4–10/sex/age/genotype). Femora and lumbar vertebrae (L_3_ to L_5_) were carefully dissected from each mouse, with the surrounding muscles removed. Epiphyses of femora were removed by scalpel, and bone marrow was flushed using RNase‐free DPBS. The intervertebral discs of L_3_ to L_5_ were gently removed. Vertebral cancellous bone, including the bone marrow, was isolated from each vertebral body by carefully removing the surrounding cortex with a scalpel under dissecting microscope while suspended in Trizol reagent (Invitrogen, Carlsbad, CA, USA). Combined femoral cortical diaphysis (left and right with no marrow) and vertebral cancellous bone (with marrow) were then immediately snap‐frozen in liquid nitrogen, separately. Frozen tissues were pulverized under liquid nitrogen with a mortar and pestle. Total RNA was extracted with the Trizol reagent (Invitrogen) according to the manufacturer's instructions. Standard cDNA was synthesized from 1 μg total RNA following the elimination of genomic DNA (QuantiTec Rev Transcription Kit, Qiagen, Hilden, Germany) and subjected to quantitative real‐time PCR (RT‐qPCR) using KAPA SYBR Fast qPCR Kits (KAPA Biosystems, Wilmington, MA, USA) using a ViiA7 Real‐Time PCR machine (Applied Biosystems, Waltham, MA, USA). Primers for tested genes were determined using the Harvard Primer Bank.^(^ ^46^ ^)^ Genes examined included those related to sex hormone enzymes and receptors (ERα, ERβ, AR, aromatase), osteoblast‐ and osteoclast‐related markers (type‐I collagen, cathepsin K, receptor activator of NF‐κB ligand [RANKL], osteoprotegerin [OPG], macrophage colony‐stimulating factor [M‐CSF], sclerostin [12 weeks only]), and reference genes (GapDH, β‐Actin) (Supplemental Table S4). GapDH and β‐Actin were used as the reference genes for the 12‐week and 30‐week groups, respectively, due to inconsistent gene expression of a single reference gene (CT values) between the genotypes in either age group (Supplemental Table S5). The relative expression level for each gene was normalized to the housekeeping genes by the cycle threshold method, △CT = CT (tested gene) – CT (housekeeping gene). In addition, fold‐change (FC) gene expression for ERβ‐dOT relative to LC was calculated using the 2^−ΔΔCt^ method.

### Serum analyses

Blood serum samples were collected by cardiac exsanguination for male and female ERβ‐dOT and LC mice at 12 weeks and 30 weeks of age (*n* = 7–10/sex/age/genotype), while mice were deeply anesthetized (4% isoflurane, 1.0 L O_2_/min). Female mice were euthanized without paying particular attention to the estrous cycle. Blood serum was isolated by centrifuge (30,000 x  *g* for 5 minutes at 4°C). Blood serum samples were analyzed for circulating sex hormone levels using a gas chromatography‐tandem mass spectrometry (GC‐MS/MS) method by Dr Claes Ohlsson's lab (Gothenburg, Sweden).^(^ ^47^ ^)^ Sex hormones analyzed included estrone (E1), estradiol (E2), testosterone (T), dihydrotestosterone (DHT), progesterone (P), and androstenedione (AE).

### Statistical analysis

Data are presented as mean ± SD. Assumptions for normality and homogeneity of variance were tested by running the Shapiro–Wilk test and Levene's test, respectively, before statistical analyses. Differences in morphological and physiological parameters (morphology, gene expression, or blood serum measures) between genotypes were analyzed via Student's *t* test (*p* < 0.05). The main effects of genotype and loading and the interaction of these factors on the adaptive response to tibial loading were determined with a linear mixed model with repeated measures,^(^ ^48^ ^)^ where the between‐subject factor was mouse genotype (ERβ‐dOT or LC), and the within‐subject factor was limb (loaded or control). When the genotype and load interaction was significant (*p* < 0.05), differences between genotypes and/or limbs were tested by pairwise comparison with Bonferroni correction. All results presented are significant unless otherwise stated. If no significant interaction was present, only the main effects were reported. Percentage differences were calculated for the effect of genotype or loading as [(ERβ‐dOT − LC) * 100 / LC] or as [(loaded – control) * 100 / control], respectively. Serum analysis to compare each circulating sex hormone level between genotypes was tested by the Mann–Whitney test (*p* < 0.05). The sex hormone levels are presented as means ± SEM. This study did not compare between sexes or ages for most outcome measures. All statistical tests were run by SPSS Statistics 26 (IBM Inc., Chicago, IL, USA).

## Results

### Mouse characteristics

In our model, ERβ mRNA levels were decreased in cortical bone of femoral diaphysis in ERβ‐dOT mice relative to the LC mice for both sexes at either age group (Supplemental Table S6), demonstrating that the conditional knockout model we used in this study represents a functional ERβ gene deletion. ERα mRNA levels were not altered with Ot‐ERβ deletion in the femoral cortical bone of male or female mice in either age group (Supplemental Table S6), confirming no compensation by increased ERα expression in osteocytes with the absence of ERβ. However, gene expression levels of AR in femoral cortical bone were 3.2‐fold higher in 12‐week‐old male ERβ‐dOT mice relative to LC (Supplemental Table S6) while not changing in 30‐week‐old male mice or female mice at either age (Supplemental Table S6). Serum analysis by GC‐MS/MS showed that all the tested circulating sex steroids (E2, E1, T, DHT, P, and AE) in female and male mice were not significantly different between ERβ‐dOT and LC mice in either age group (Supplemental Table S7), demonstrating that neither systemic sex steroid levels nor negative feedback regulation of the serum sex steroid was interrupted by the conditional knockout of Ot‐ERβ. Male mice, at both ages, showed undetectable levels of E1 and E2, so although some small changes may have occurred in these hormones, the effects could not be detected here. It is important to note that, in our study, the estrous cycle of female mice was not tracked for blood serum collection. Regardless, even given the amount of variation caused by using this approach, both young and adult females still show similarity across all hormones measured (Supplemental Table S7*B*).

The ERβ‐dOT mice, neither male nor female, showed no differences from the LC mice in terms of body weight (Supplemental Table S8) or tibial length (Supplemental Table S9) at 10 weeks or 28 weeks of age. After 2 weeks of loading, no significant changes in body weight appeared in 12‐week‐old or 30‐week‐old male and female mice (Supplemental Table S8). In addition, the loaded and control limbs did not differ in tibial length between genotypes, regardless of sex or age (Supplemental Table S9).

### Sex‐dependent changes in bone morphology with Ot‐ERβ deletion in 12‐week‐ and 30‐week‐old mice

**Fig. 1:**
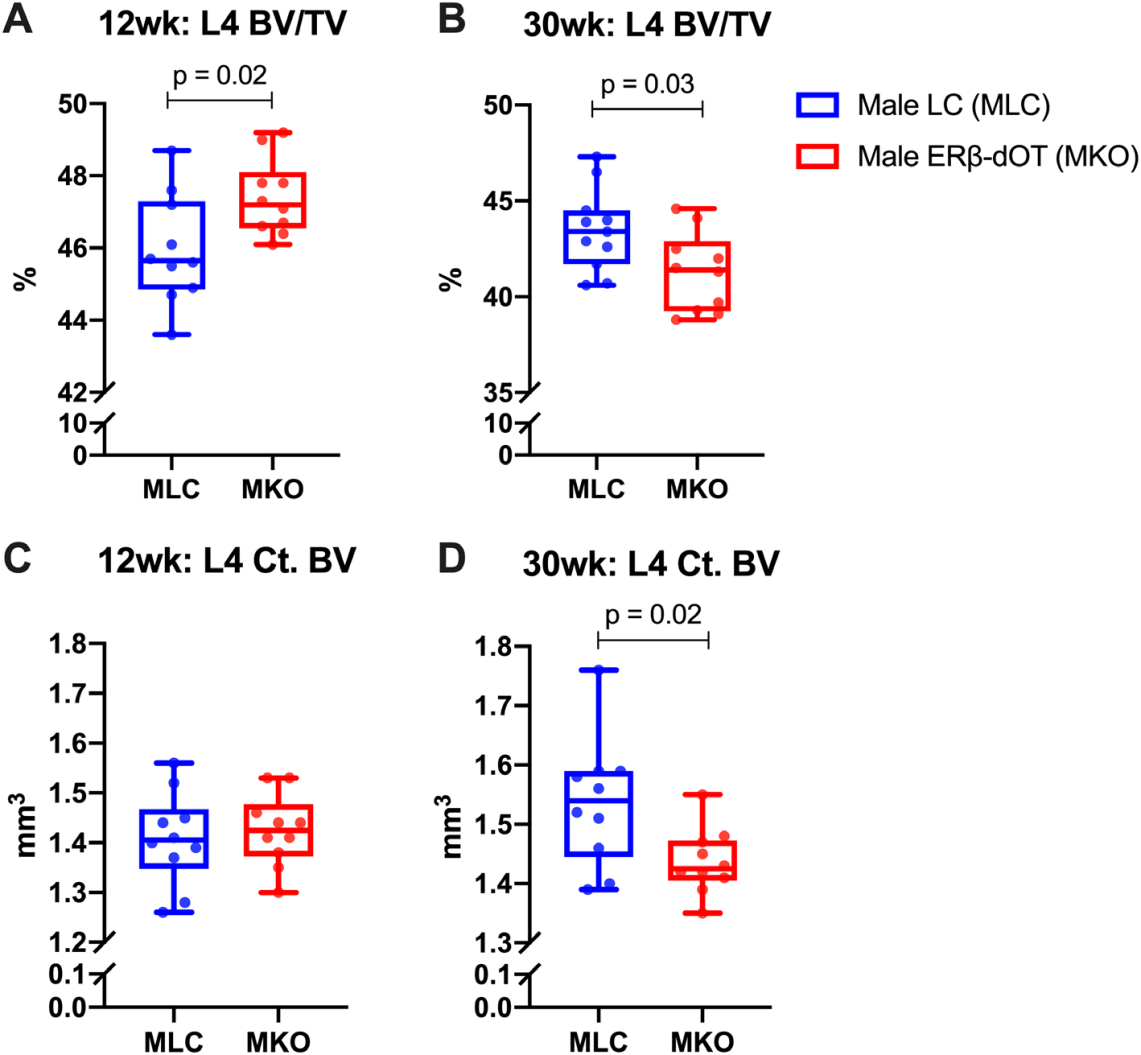
Bone morphology analysis of L_4_ in 12‐week‐old (young) and 30‐week‐old (adult) male littermate controls (LC) and ERβ‐dOT (knockout [KO]) mice. The lumbar vertebral body (L_4_) of male LC (MLC, blue) and ERβ‐dOT (MKO, red) mice at 12 weeks (*A*, *C*) and 30 weeks of age (*B*, *D*) were analyzed by micro‐CT. L_4_ trabecular bone volume fraction (BV/TV; *A*, *B*) and cortical bone volume (Ct.BV; *C*, *D*) of 12‐week‐old and 30‐week‐old MLC and MKO are presented as box plots with median and interquartile ranges (IQR; 25th to 75th percentile) including all data points (*n* = 10–12 per group). Significant differences in L_4_ bone morphology between genotypes were tested by Student's *t* test (*p* < 0.05). Specific *p* values are shown when *p* < 0.05.

Bone morphology analysis by micro‐CT showed that 12‐week‐old male ERβ‐dOT mice had increased cancellous bone mass (BV/TV: +3.1%) in L_4_ compared with LC mice (Fig. 1*A*), principally owing to increased Tb.N (+7.6%) and Tb.BV (+11.7%) (Supplemental Table S10). Consistent with the increased L_4_ BV/TV, the 12‐week‐old male ERβ‐dOT mice had greater L_4_ Tb.BS (+8.2%) compared with LC mice (Supplemental Table S10). No difference in L_4_ cortical volume occurred between genotypes in male mice at 12 weeks old (Fig. 1*C*). In contrast, adult male ERβ‐OT at 30 weeks old had reduced cancellous (BV/TV: −5.0%; Fig. 1*B*; Supplemental Table S10) and cortical bone (Ct. BV: −6.5%; Fig. 1*D*) in L_4_ relative to LC mice. Reduced Tb.BV (−8.6%) resulted from decreased Tb.N (−6.3%) and Tb.Th (−3.4%) relative to adult LC mice (Supplemental Table S10). Adult male ERβ‐dOT mice showed a reduced trabecular bone surface (Tb.BS: −6.5%) in L_4_ compared with LC mice (Supplemental Table S10). However, no morphological changes appeared in the tibial cortical or cancellous bone in 12‐week‐ or 30‐week‐old male mice with Ot‐ERβ deletion (Supplemental Table S11). Female ERβ‐dOT and LC mice in both age groups showed no difference in cortical or cancellous bone mass in L_4_ (Supplemental Table S10). For the tibias in female mice, no difference in cortical or cancellous bone was caused by the Ot‐ERβ deletion, except a decrease in bone mineral density in the midshaft tibial cortical bone (Ct. BMD: −5.7%) of the 12‐week‐old female ERβ‐dOT compared with the LC mice (Supplemental Table S11). Together, these results show that Ot‐ERβ regulates bone morphology differently by sex, age, and bone compartment. Vertebral bone mass is increased in young male mice (cancellous only) but decreased in adult male mice (cortical and cancellous bone) by the deletion of Ot‐ERβ.

### Sex‐dependent changes in the tibial adaptive response to mechanical loads with Ot‐ERβ deletion in 12‐week‐ and 30‐week‐old mice

**Fig. 2:**
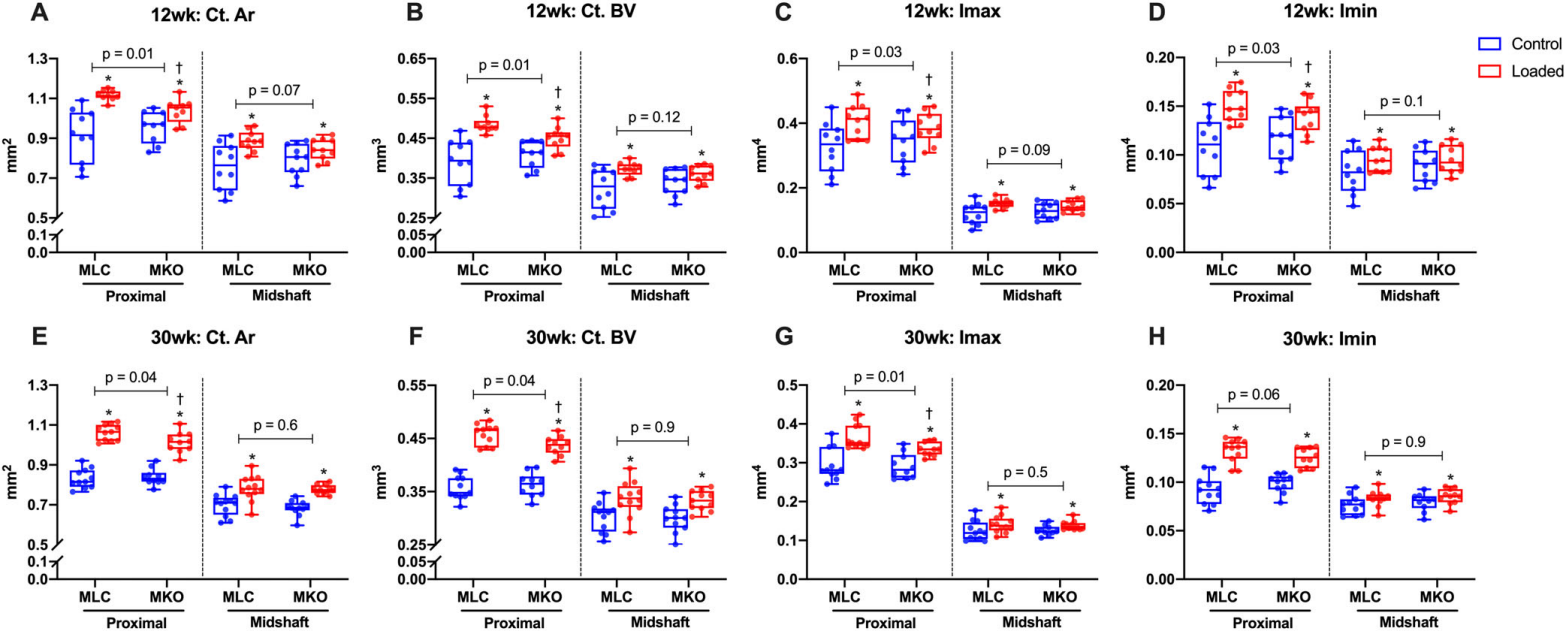
Tibial cortical adaptive response to mechanical loading in 12‐week‐old (young) and 30‐week‐old (adult) male littermate controls (LC) and ERβ‐dOT (knockout [KO]) mice. Load‐induced cortical adaptive response was determined after 2 weeks of loading by micro‐CT analysis of the proximal (37%) and at the midshaft (50%) of loaded (red) and control (blue) tibias of male LC (MLC) and ERβ‐dOT (MKO) mice at 12 weeks (*A–D*) and 30 weeks of age (*E–H*). Data are presented as box plots with median and interquartile ranges (IQR; 25th to 75th percentile) including all data points (*n* = 10–12 per group). (*A*, *E*) Cortical area (Ct.Ar). (*B*, *F*) Cortical bone volume (Ct.BV). (*C*, *G*) Maximum moment of inertia (Imax). (*D*, *G*) Minimum moment of inertia (Imin). The effects of genotype and load and their interaction were tested by the linear mixed model with repeated measures followed by pairwise comparisons with Bonferroni correction. *p < 0.05 for significant loading effect (loaded versus control). Specific *p* values are presented in the figure to show the significant genotype effect on the loading response (*p* < 0.05). ^†^ *p* < 0.05 for the significant difference in the loaded limbs between genotypes by the linear mixed model with repeated measures.

**Fig. 3:**
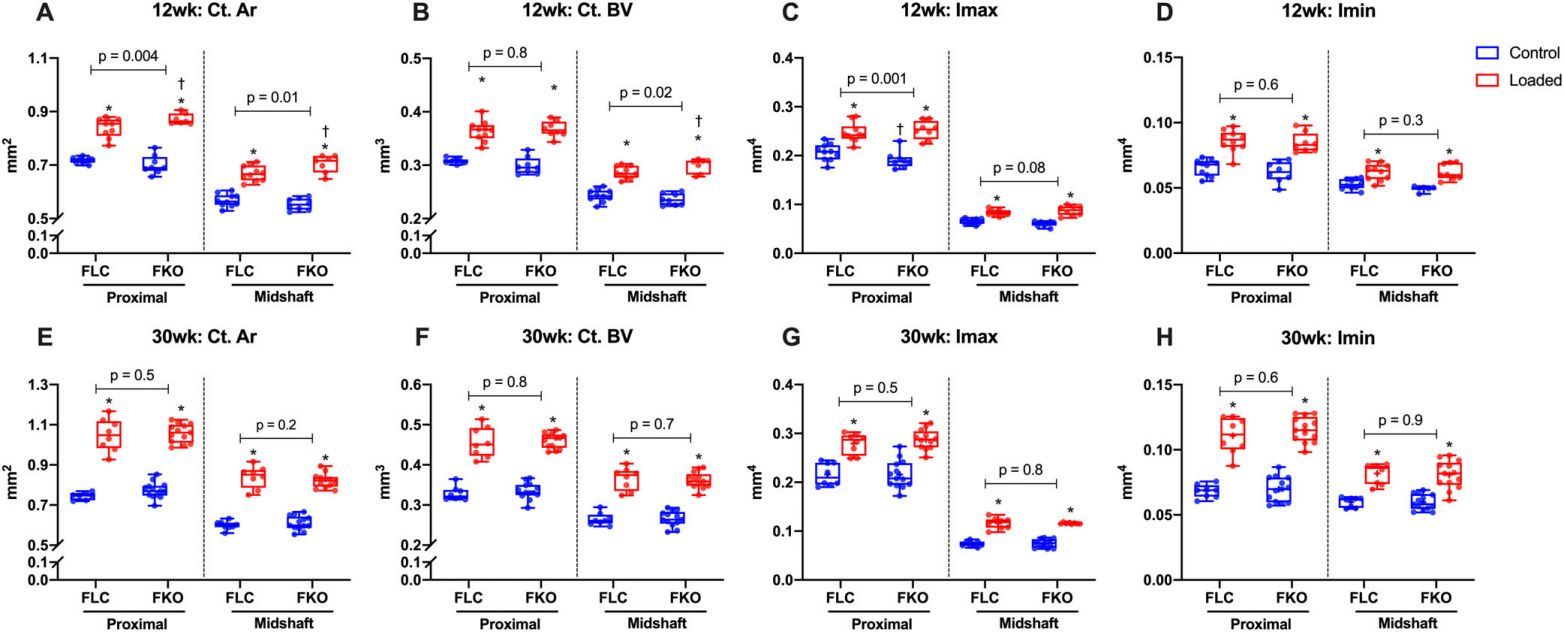
Tibial cortical adaptive response to mechanical loading in 12‐week‐old (young) and 30‐week‐old (adult) female littermate controls (LC) and ERβ‐dOT (knockout [KO]) mice. Load‐induced cortical adaptive response was determined after 2 weeks of loading by micro‐CT analysis of certain volumes of interest (VOIs) from the proximal metaphysis (37%) and at the mid‐diaphysis (50%) of loaded (red) and control (blue) tibias of female LC (FLC) and ERβ‐dOT (FKO) mice at 12 weeks (*A–D*) and 30 weeks of age (*E–H*). Data are presented as box plots with median and interquartile ranges (IQR; 25th to 75th percentile) including all data points (*n* = 10–12 per group). (*A*, *E*) Cortical area (Ct.Ar). (*B*, *F*) Cortical bone volume (Ct.V). (*C*, *G*) Maximum moments of inertia (Imax). (*D*, *G*) Minimum moments of inertia (Imin). The effects of genotype and load and their interaction were tested by the linear mixed model with repeated measures followed by pairwise comparisons with Bonferroni correction. *p < 0.05 for significant loading effect (loaded versus control). Specific *p* values are presented in the figure to show the significant genotype effect on the loading response (*p* < 0.05). ^†^ *p* < 0.05 for the significant difference in the loaded limbs between genotypes by the linear mixed model with repeated measures.

After 2 weeks of compressive loading, the loaded tibias showed a significant osteogenic adaptation to mechanical loading in both cortical and cancellous bone compared with the control tibias in male and female ERβ‐dOT and LC mice at both ages (Supplemental Tables S12 and S13). Male mice with Ot‐ERβ deletion at 12 weeks and 30 weeks old presented a reduced cortical osteogenic response in the loaded tibias compared with the LC mice (Fig. 2). Load‐induced increases in Ct.Ar, Ct.BV, Imax, and Imin (12 weeks only) in the proximal tibias (37%) were diminished in male ERβ‐dOT mice at 12 weeks and 30 weeks of age relative to LC mice (Fig. 2; Supplemental Table S12). However, the tibial midshaft (50%) in male ERβ‐dOT mice was similar to LC mice at both ages in response to mechanical loading (Fig. 2*A*, *D*). Female ERβ‐dOT mice at 12 weeks old showed an enhanced load‐induced cortical osteogenic response, with greater Ct.Ar, Ct.BV, Imax, and Ct.BMD, at proximal (37%) and midshaft (50%) tibias (Fig. 3; Supplemental Table S13*A*). Deletion of Ot‐ERβ did not affect the cortical osteogenic response in the tibias of 30‐week‐old female mice (Fig. 3*E–H*; Supplemental Table S13*B*). The load‐induced adaptive response of cancellous bone in the tibial proximal metaphysis showed no differential effects with Ot‐ERβ deletion in male or female mice in either age group (Supplemental Tables S12 and S13; Supplemental Fig. S1). Taken together, these results show that Ot‐ERβ mediates load‐induced tibial adaptation differently by sex and bone compartment. The osteogenic adaptation of cortical bone is reduced in male mice (young and adult) but enhanced in female mice (young group only) by the absence of Ot‐ERβ. However, cancellous bone adaptation in response to mechanical loading is not affected by the deletion of Ot‐ERβ for male and female mice of both ages.

### Changes in gene expression with Ot‐ERβ deletion in the skeletons of male and female mice

**Fig. 4:**
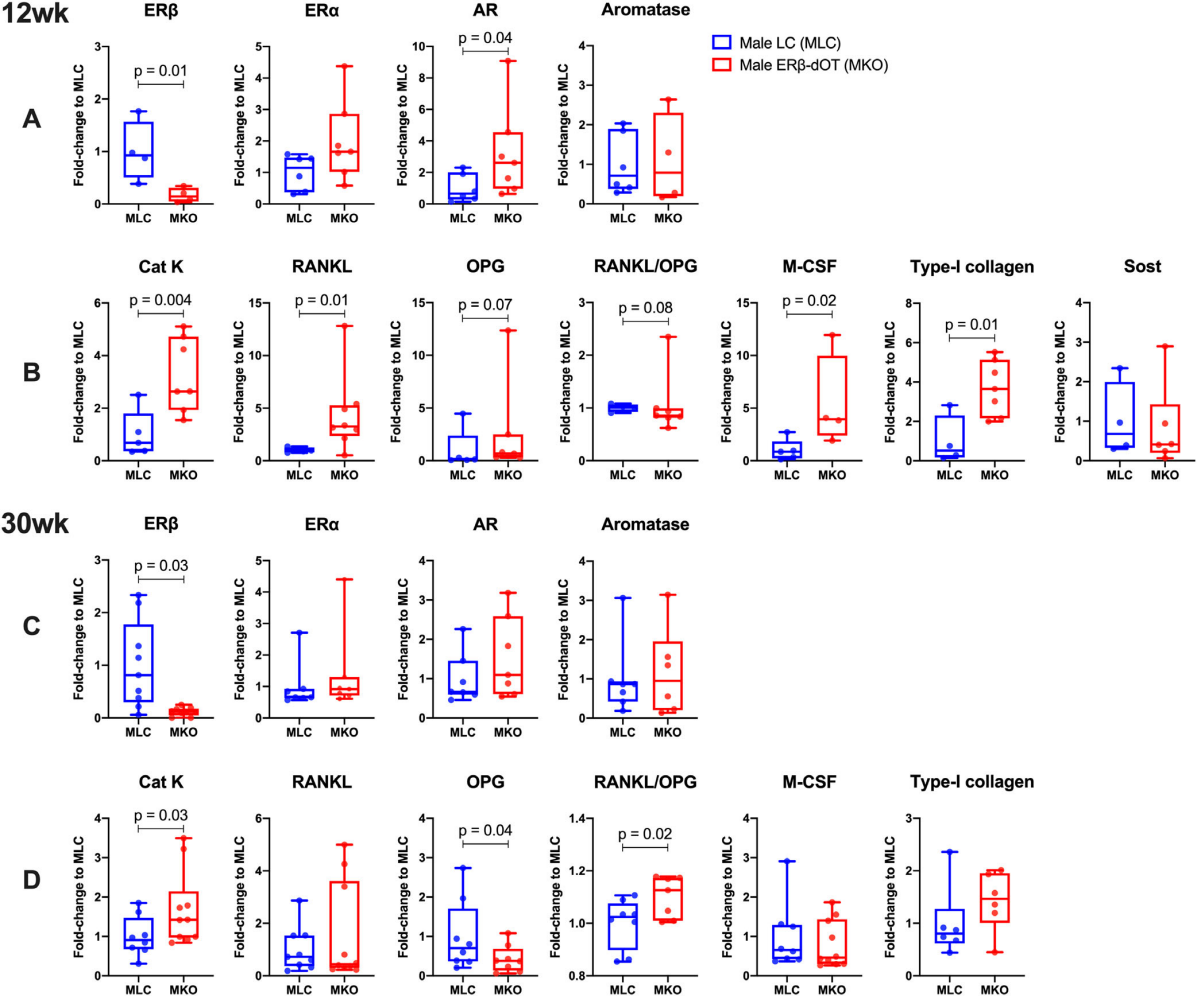
Fold‐change of gene expressions in femoral cortical bone for 12‐week‐old (*A, B*) and 30‐week‐old (*C, D*) male ERβ‐dOT (knockout [KO]) mice relative to the corresponding male littermate controls (LC) mice. Total RNA was extracted from femoral cortical bone (no marrow) of male LC (MLC, blue) and ERβ‐dOT (MKO, red) mice at 12 weeks and 30 weeks of age. Relative values of the cycle threshold (delta Ct) were calculated relative to the reference genes. Fold‐change (FC) of gene expression in the tibial cortical bone of MKO was normalized to MLC (FC: 1.0) and is presented as box plots with median and interquartile ranges (IQR; 25th to 75th percentile) including all data points (*n* = 4–10 per group). Sclerostin expression was only tested in young MLC and MKO (*B*). The fold‐change of RANKL/OPG ratio (*B*, *D*) was calculated as △RANKL/△OPG using delta CT values and normalized to MLC (FC: 1.0). Significant difference in gene expression between genotypes was tested at the delta CT level by Student's *t* test (*p* < 0.05). Specific *p* values are shown when 0.001 < *p* < 0.1. NS = no significant difference between genotypes (*p* > 0.1).

Ot‐ERβ was shown to play an opposing role in maintaining the vertebral cancellous bone for young (12 weeks) and adult (30 weeks) male mice. To determine the potential cellular mechanisms for this age‐related regulation of Ot‐ERβ, gene expression of key markers were tested by qPCR. Femoral cortical bone of young male ERβ‐dOT mice showed upregulated mRNA levels of type‐I collagen (FC: 3.7), Cat K (FC: 3.3), M‐CSF (FC: 5.4), and RANKL (FC: 4.4) relative to LC mice (Fig. 4*A*, *B*; Supplemental Table S6). Additionally, trends for altered expression of OPG (*p* = 0.07) and RANKL/OPG levels (*p* = 0.08) were shown in young male ERβ‐dOT mice (Fig. 4*B*; Supplemental Table S6). Cortical bone samples for adult ERβ‐dOT mice showed upregulated Cat K (FC: 1.7) expression and an elevated ratio of RANKL/OPG, resulting from downregulated OPG levels (FC: 0.4) relative to LC mice (Fig. 4*C*, *D*; Supplemental Table S6). Bone formation marker, type‐I collagen, was not altered in older ERβ‐dOT male mice (Fig. 4*D*; Supplemental Table S6). No difference in aromatase expression was present between ERβ‐dOT and LC male mice at either age (Fig. 4*A–D*; Supplemental Table S6). In contrast, female mice at both ages showed no change in gene expressions for bone resorption‐ or formation‐related markers in the femoral cortical bone between ERβ‐dOT and LC mice (Supplemental Table S6). Moreover, the tested genes in L_4_ to L_6_ cancellous bone (with marrow) were not differentially expressed between ERβ‐dOT and LC mice at either sex or age group, which is likely due to the overwhelming gene expression in the bone marrow cells relative to the osteocytes and bone lining cells in vertebral cancellous bone (Supplemental Table S14). Overall, in femoral cortical bone, bone resorption‐related (Cat K, RANKL, M‐CSF) and bone formation‐related (type‐I collagen) markers, as well as AR, are elevated in young male ERβ‐dOT mice relative to LC. Similarly, upregulated Cat K and downregulated OPG with increased RANKL/OPG levels are observed in the femoral cortical bone of adult male mice lacking Ot‐ERβ. However, relevant changes are not shown in the femoral cortical bone of young and adult female mice.

## Discussion

Our study investigates the role of ERβ in osteocyte‐mediated regulation of bone mass maintenance and skeletal adaptive response to mechanical stimuli in male and female mice with aging. Deletion of Ot‐ERβ leads to opposing effects on skeletal morphology in young (12 weeks) and adult (30 weeks) male mice. Cancellous bone was increased in the L_4_ of young male ERβ‐dOT mice compared with the LC. However, cortical bone was unaltered between genotypes. The adult male ERβ‐dOT mice had reduced bone mass in L_4_ cortical and trabecular bone relative to the LC. In addition, L_4_ bone morphology, for both the cortical and cancellous bone, in female mice at either age was not altered by the deletion of Ot‐ERβ. This differing effect of Ot‐ERβ deletion on bone mass suggests that Ot‐ERβ mediates bone morphology in male but not female mice, and its action on male mouse skeleton varies by age and compartment. However, tibial bone morphology was not affected by the absence of Ot‐ERβ in male or female mice at either age. As it is suggested that ERβ may primarily regulate cancellous bone (re)modeling,^(^ ^49^, ^50^ ^)^ the morphological impact of Ot‐ERβ deletion may be more pronounced in the vertebrae than in the tibia, which has relatively little cancellous bone.

The age‐specific imbalance in vertebral bone mass in male ERβ‐dOT mice was examined in relation to gene expression using femoral cortical bone. Increased bone turnover in the young male ERβ‐dOT mice was indicated by upregulated mRNA levels for bone formation (type‐I collagen) and bone resorption markers (RANKL, M‐CSF, Cat K) relative to the LC mice. Additionally, patterns for OPG and RANKL/OPG ratio in young male ERβ‐dOT mice were not statistically significant, and results regarding these genes remain inconclusive at this time. Adult ERβ‐dOT male mice may likely have increased osteoclastogenesis and osteoclast activity, as demonstrated by elevated Cat K levels and reduced OPG levels with increased RANKL/OPG ratio. Although further static or dynamic histomorphometry was not conducted in this study, we would hypothesize that young male mice subjected to Ot‐ERβ deletion might experience greater overall bone turnover, leading to increases in bone mass. In contrast, adult male ERβ‐dOT mice might undergo elevated bone resorption with unaltered bone formation, consistent with the decreased vertebral bone mass relative to LC. Thus, Ot‐ERβ may regulate the age‐specific bone turnover in male mice via different mechanisms, that it inhibits bone turnover in young male mice by reducing bone formation and osteoclastogenesis but protects bone morphology in adult male mice by suppressing the generation and the resorptive activity of mature osteoclasts. However, at this time, these conclusions remain hypothetical without additional work using serum‐based resorption and formation markers (CTX, P1NP) or histomorphometry.

ERβ has been implied to mediate bone maintenance through interacting with ERα.^(^ ^14^, ^15^, ^23^, ^26^ ^)^ Several global KO studies have discussed the suppressive effect of ERβ on ERα levels in bone tissue.^(^ ^18^, ^26^, ^51^, ^52^ ^)^ Besides, ERs and AR have been reported to form heterodimers for activation by testosterone and estrogen.^(^ ^1^, ^14^, ^53^ ^)^ However, most previous studies were performed in female mice, assuming that estrogen receptors have a greater impact on female but not male skeletons,^(^ ^11^, ^23^, ^24^, ^27^ ^)^ although clinical studies have shown that ERs also play significant roles in periosteal expansion and osteoprotective regulation in males.^(^ ^1^, ^17^ ^)^ There were two global KO studies examining the skeletal role of ERβ in male mice, but they showed different results.^(^ ^25^, ^27^ ^)^ One study concluded that ERβ might protect the mineral density of the cortical and trabecular bone in male mice (18 weeks) by preserving bone formation,^(^ ^25^ ^)^ whereas the other reported that no skeletal phenotypic alternation appeared in male mice (1 year old) with global ERβ deletion.^(^ ^27^ ^)^ The inconsistent results might come from the differences in age and genetic mouse strain or the potential endocrine disturbance caused by the global KO. Additionally, many of these early hypotheses are based on global ERβ or ERαβ KO models that caused elevated circulating IGF‐I and estrogen levels^(^ ^21^, ^26^ ^)^ or increased fat mass,^(^ ^25^ ^)^ which make it difficult to discern the involvement of ERβ in the direct regulation of bone mass.

To address whether the effect of Ot‐ERβ deletion on bone morphology could be associated with subsequent changes in other sex steroid receptors, we also measured the local mRNA level of ERα and AR in the femoral cortical bone. Contrary to previous global KO studies, the ERα mRNA levels were not altered by Ot‐ERβ deletion in the femoral cortical bone of male and female mice of both ages, suggesting that Ot‐ERβ might not regulate the expression or function of ERα in bone. However, gene expression of AR was upregulated in femoral cortical bone in young, but not adult, male mice with Ot‐ERβ deletion, suggesting the osteoprotective actions of testosterone and estradiol might be facilitated through increased AR number. A prior immunohistochemistry study on the papillary dermis of human skin reported the regulation of ERβ in suppressing AR expression through binding to estradiol.^(^ ^14^, ^54^ ^)^ While targeting connective skin tissue (dermis), this study is consistent with our findings in skeletal connective tissue that Ot‐ERβ has an inhibitory role on AR levels in young male mice skeletons. Furthermore, AR deletion in osteoblasts and osteocytes led to cortical bone loss in young and adult male mice (6 – 32 weeks) by disrupting bone remodeling,^(^ ^55^, ^56^ ^)^ suggesting the osteoprotective role of AR in male bone mass regulation. Thus, the overexpression of AR caused by Ot‐ERβ deletion in our young male mice might induce osteoblast activities and promote bone formation, which would also help to explain the opposing effect of Ot‐ERβ deletion on bone mass between young and adult male mice. However, neither Ot‐AR deletion nor AR protein expression was conducted in our study. Therefore, the direct explanation for the causal linkage between ERβ and AR in bone formation remains undetermined. Future immunohistochemistry studies are needed to investigate the role of ERβ/AR interaction in osteocytes. In addition, AR is a receptor of testosterone and DHT but can also interact with estrogen indirectly by forming AR‐ER heterodimers or AR homodimers.^(^ ^1^, ^14^ ^)^ Therefore, we hypothesize that the overexpression of AR transcript level might potentially contribute to the osteoprotective regulations of both estrogen and testosterone, resulting in increased bone mass in young male ERβ‐dOT mice. Future *in vitro* work on osteocytes with AR‐ERβ deletion is needed to verify this conjecture. Alternatively, it is also possible that the increased AR level in the femoral cortical bone of young male ERβ‐dOT mice results from a heterogeneous cell population in the RNA extraction. Tissue collected from the femoral cortical diaphysis of young male mice contained osteocytes but also a mixture of cells from the endosteum and periosteum (stromal cells, osteoblasts, osteoclasts, and bone lining cells) that could be responsible for the increased AR levels in young male mice with Ot‐ERβ deletion. Spatial transcriptomics, which provides tissue‐ and location‐specific assessment, might be used to better detect osteocyte gene expressions in cortical bone.

In female mice, the mRNA levels of Cat K and type I collagen, as well as the ERα and AR, in the femoral cortical bone were not altered by Ot‐ERβ deletion, suggesting that, in light of similar micro‐CT results in ERβ‐dOT and LC mice, Ot‐ERβ might not directly mediate osteoblast or osteoclast function in bone remodeling for female mice. Additionally, mRNA levels of the aromatase in male and female femoral cortical bone showed no difference between ERβ‐dOT and LC for both age groups, indicating the aromatization and local levels of testosterone and estradiol in bone tissue might not vary with Ot‐ERβ deletion.

Conditional deletion of ERs in osteoblasts and osteocytes has been induced using several Cre/lox models.^(^ ^29^, ^30^ ^)^ It has been recognized that the roles of ERs in skeletal growth and remodeling vary between the different stages of osteoblastogenesis. Deletion of ERα in osteoprogenitors by *Prx1/Osx1*‐Cre has shown that at this early stage in osteoblastogenesis, ERα protects cortical bone in growing male and female mice, with no effect on cancellous bone.^(^ ^57^ ^)^ However, in female mice, ERβ in osteoprogenitors (*Prx1*‐Cre) was shown to inhibit cancellous bone growth, with no effect on cortical bone.^(^ ^58^ ^)^ Thus, ERα and ERβ in pre‐osteoblasts mediate bone growth in different bone tissues in opposite ways. The localized cortical and cancellous results of these studies agree with immunohistochemistry studies showing that ERα is primarily expressed in osteoblasts and osteocytes of cortical bone, whereas ERβ expression is more strongly expressed in the cancellous bone.^(^ ^49^, ^50^ ^)^ In addition, ERα deletion in mature osteoblasts/osteocytes by osteocalcin‐Cre (*OC*‐ERα) revealed the protective regulation of *OC*‐ERα in the cortical and cancellous bone of both growing (12–18 weeks) and adult (28 weeks) female mice.^(^ ^5^, ^59^, ^60^ ^)^ The relevant role of ERα in male mice remains inconclusive, possibly because of differences in how the floxed mouse models in these studies were produced. However, ERβ has not been pursued in derived osteoblasts/osteocytes using the *OC*‐Cre model. Therefore, more work needs to be conducted at this cell stage to understand the role of ERβ in both osteoblasts and osteocytes.

Our study is the first to examine the *in vivo* deletion of ERβ in osteocytes (*Dmp1*‐Cre) for bone maintenance in female and male mice of different age groups. The inhibitory regulation of Ot‐ERβ in vertebral cancellous bone in our young male mice (12 weeks) is contrary to the role of Ot‐ERα (*Dmp1*‐Cre) in cancellous bone protection reported in growing male mice (11 weeks),^(^ ^32^ ^)^ suggesting that Ot‐ERα (supportive) and Ot‐ERβ (inhibitory) play opposing roles in cancellous bone maintenance in male mice during early growth. Interestingly, the inhibitory regulation of Ot‐ERβ in the skeleton shifts to a protective effect in mature male mice (30 weeks). However, no Ot‐ERα or Ob‐ERβ study has reported results in mature male mice, which makes the relationship between Ot‐ERα and Ot‐ERβ in adult male mice inconclusive at this time. Similar to our findings, Ot‐ERα has no effect on cortical bone morphology in young female mice (12 weeks),^(^ ^31^ ^)^ suggesting a minor role of both Ot‐ERs in cortical bone maintenance for female mice. In growing females (12 weeks), Ot‐ERα has been shown to protect cancellous bone by regulating bone formation.^(^ ^31^ ^)^ Collectively, ERα and ERβ in osteocytes perform different functions in bone maintenance, varying between age, sex, and bone compartment. ERα in osteocytes likely plays a major role in bone mass protection, especially for cancellous bone, in female mice. In contrast, ERβ might play a dispensable role in female mouse cortical and cancellous bone metabolism but is important for male cancellous bone, although its regulation varies with age. Further studies of conditional KO models of ERα, ERβ, and ERα/β at different ages, combined with immunoassay and histological analyses, are needed to provide additional evidence to reveal the relationship between ERα and ERβ in bone mass protection.

Consistent with the effect of ERβ on bone maintenance, there was also a sex‐dependent effect of Ot‐ERβ on the cortical adaptive response to tibial compressive loading. Our findings indicate that Ot‐ERβ is required to achieve the load‐induced anabolic response of tibial cortical bone (37%) for male mice in both age groups. Contrary to the results for male mice, Ot‐ERβ plays a suppressive role in the cortical adaptation of tibias (37% and 50%) in young female mice. However, no relevant difference was shown in adult female mice, indicating that ERβ might exert an age‐specific role in cortical bone adaptation for female mice. Alternatively, the similar osteogenic cortical response to load in the adult female mice might result from the high strains (~1800 με) induced by the applied load (–11 N), which may lead to a maximum osteogenic adaptation and could potentially hide any genotypic impact.^(^ ^61^ ^)^ The cancellous adaptive response was not affected by the Ot‐ERβ deletion in male and female mice at both ages, suggesting a dispensable role for Ot‐ERβ in mediating the load‐induced structural adaptation of cancellous bone. Collectively, our tibial loading study indicates that Ot‐ERβ likely does not mediate load‐induced cancellous adaptation in male or female mice at either age while still exerting a sex‐dependent regulation of cortical adaptation.

This differential regulation of Ot‐ERβ in skeletal adaptation may be attributed to the interaction of ERα and ERβ in mechanotransduction. Earlier *in vivo* global KO studies indicated that ERα is primarily responsible for skeletal adaptation in female mice.^(^ ^11^, ^12^, ^13^, ^18^, ^62^ ^)^ *In vitro* studies showed that ERα is required for cell proliferation induced by applied strains,^(^ ^19^, ^20^ ^)^ and this ERα‐regulated mechano‐responsiveness was elevated in osteoblast‐like cells lacking ERβ,^(^ ^20^ ^)^ suggesting the inhibitory effect of ERβ on ERα actions in response to mechanical stimuli. Similar to our results, greater cortical adaptation was induced by loading in female mice (16–17 weeks) with systemic ERβ deletion.^(^ ^11^, ^25^ ^)^ Thus, the inhibitory regulation of Ot‐ERβ in cortical bone adaptation for our young female mice might occur by inhibiting ERα‐mediated actions on skeletal mechanotransduction. Additional *in vivo* studies with conditional ERα deletion showed that in young and adult female mice (10–12 weeks and 28 weeks), it is Ob‐ERα, rather than Ot‐ERβ, that likely mediates the skeletal osteogenic response.^(^ ^5^, ^12^, ^62^ ^)^ This further implies that in female mice skeletons, bone cells might react to mechanical signals through different ERs, where mechanotransduction in osteocytes is dominated by ERβ relative to ERα, whereas the mechanoadaptation in osteoblasts may rely upon ERα. Future studies with conditional dual deletions of Ob‐ERα and Ot‐ERβ may help to prove our hypothesis and reveal the relationship of ERα and ERβ in skeletal mechanotransduction in female mice. Very few studies have investigated the role of ERs in skeletal mechanoadaptation in male mice. ERα was indicated to have a negligible effect on load‐induced cortical adaptation in male mice,^(^ ^62^, ^63^, ^64^ ^)^ suggesting that ERβ may dominate this role. An *in vivo* study using male mice with global ERβ KO showed reduced cortical osteogenic adaptation,^(^ ^11^ ^)^ contradicting our findings for Ot‐ERβ, which is likely due to the use of a global KO model. By far, our study is the only *in vivo* study demonstrating the local role of Ot‐ERβ in mediating the adaptive response to mechanical stimulus in male mice skeletons.

It is also necessary to consider the role of sex hormones in load‐induced skeletal adaptation as the mechano‐responsiveness reduces in both human and mouse skeletons with aging.^(^ ^3^, ^4^ ^)^ Further studies examining sex hormone removal and supplementation could be valuable in regard to determining the ligand dependency of ERβ on the skeletal response to mechanical loading. In addition, we know ERs and AR are both important in modulating skeletal adaptation in response to loading.^(^ ^10^, ^19^, ^32^, ^64^, ^65^ ^)^ The function and expression of ERs can be induced by mechanical signals through ligand‐independent pathways.^(^ ^10^, ^12^, ^19^, ^51^ ^)^ Increased ERα mRNA level by mechanical stimuli was detected in previous *in vivo* and *in vitro* loading studies.^(^ ^51^, ^52^ ^)^ However, very few studies have conducted transcriptomic or proteomic analysis on loaded limbs, especially in regard to hormone receptors.^(^ ^66^, ^67^, ^68^ ^)^ Unfortunately, ER or AR mRNA levels or protein contents for these receptors were not examined in our loaded tibiae. Expression of changes in these sex hormone receptors with mechanical loading could be addressed in future studies to tackle the underlying signaling mechanisms between ERα, ERβ, and AR during skeletal adaptation in response to mechanical stimuli.

Our study is constrained by additional limitations. First, gene expression analyses in the femoral cortical bone are presented as a proxy for the axial and appendicular skeleton. Attempts were made to isolate trabecular bone from the vertebrae, but the number of marrow cells in the samples made it difficult to provide clear results, as even ERβ did not differ in the cancellous vertebral samples between the two genotypes examined. If we had been able to use different capture techniques, like laser capture microdissection or making more intricate dissections to isolate cancellous bone,^(^ ^68^, ^69^ ^)^ we might have been able to test vertebral‐specific osteocyte data. Another limitation of this study is the lack of dynamic histomorphometry analysis. While gene expression data from femoral samples provided insight into the regulatory effects of Ot‐ERβ deletion in cortical and cancellous tissues, immunohistochemical and histomorphometry measures of receptor expression (ERα, ERβ, AR) and osteoblast/osteoclast activities in the vertebrae and tibias would provide additional information on ERβ's role in skeletal growth and (re)modeling during aging and in response to mechanical loading. In addition, our results of Ot‐ERβ are based on the work in a mouse model. Limitations may exist in translating the results from mice to humans due to the differences in the mouse skeleton relative to humans, for instance, the lack of osteonal remodeling, continuous bone growth after puberty, maintained estrogen and testosterone levels after aging, and the lack of true menopause in mice.^(^ ^70^ ^)^ Lastly, our study used the *DMP1*‐Cre model to achieve the conditional knockout of Ot‐ERβ. Although mature osteoblasts expressing *DMP1* are shown to be destined to differentiate into osteocytes,^(^ ^35^ ^)^ the potential deletion of ERβ in late‐stage osteoblasts might still contribute to our results. We must also consider that *DMP1* is not only present in late osteoblasts and osteocytes^(^ ^71^ ^)^ but also has been found in soft tissues such as skeletal muscle, brain, pancreas, and kidneys.^(^ ^72^, ^73^ ^)^ Certainly, the expression of *DMP1* and its deletion of ERβ in skeletal muscle and kidneys should be examined in further studies using the *Dmp1*‐8 kb‐Cre in the skeleton.

In conclusion, for the first time, our study reveals the sex‐dependent roles of ERβ in osteocyte‐regulated bone turnover in young and adult mice. While Ot‐ERβ plays a minor role in female mice skeletons, it regulates cancellous bone morphology in male mice differently by age. Ot‐ERβ maintains cancellous bone mass for young male mice but protects cortical and cancellous bone in adult male mice. When tibial mechanical loading is applied, Ot‐ERβ mediates the mechanoadaptation of cortical but not cancellous bone differently by sex. Ot‐ERβ is required in young and adult male mice but plays an inhibitory role in young female mice during cortical mechanoadaptation. Given further studies on the interaction of ERβ with other hormone receptors and in the absence of sex hormones, we hope we can provide a pathway for discovering additional therapeutic anabolic avenues in helping to prevent osteoporosis. With the direct evidence of Ot‐ERβ, we hope to elucidate the function and relationship of ERs in osteocyte regulation of bone (re)modeling during growth, aging, and mechanobiology.

## Author Contributions

**Xiaoyu Xu:** Conceptualization; data curation; formal analysis; investigation; methodology; project administration; validation; visualization; writing – original draft; writing – review and editing. **Haisheng Yang:** Conceptualization; data curation; formal analysis; investigation; methodology; software; writing – review and editing. **Whitney A. Bullock:** Conceptualization; investigation; project administration; supervision; writing – review and editing. **Maxim A. Gallant:** Data curation; formal analysis; investigation; methodology; project administration; resources; writing – review and editing. **Claes Ohlsson:** Conceptualization; investigation; methodology; resources; writing – review and editing. **Teresita M. Bellido:** Conceptualization; project administration; writing – review and editing. **Russell P. Main:** Conceptualization; data curation; formal analysis; funding acquisition; investigation; methodology; project administration; resources; software; supervision; validation; visualization; writing – original draft; writing – review and editing.

## Conflict Of Interest

The authors declare that there is no conflict of interest that could be perceived as prejudicing the impartiality of the research reported.

### Peer Review

The peer review history for this article is available at https://publons.com/publon/10.1002/jbmr.4731.

## Supporting information


**Supplemental Fig. S1.** Tibial cancellous adaptive response to the compressive loading in 12‐week‐old (young) and 30‐week‐old (adult) male and female LC and ERβ‐dOT (KO) mice. The structural adaptive response in cancellous bone was determined in the loaded (Loaded, red) and controlled (Control, blue) tibias of male and female LC (MLC, FLC) and ERβ‐dOT (MKO, FKO) mice at 12 weeks (*A*, *B*) and 30 weeks of age (*C*, *D*) by micro‐CT analysis. Trabecular bone volume fraction (BV/TV) of the proximal tibia is shown. Data are presented as box plots with median and interquartile ranges (IQR; 25th to 75th percentile) including all data points (*n* = 10–12 per group). The effects of genotype and load and their interaction were tested by the linear mixed model with repeated measures followed by pairwise comparisons with Bonferroni correction. *p < 0.05 for significant loading effect for the same genotype by the linear mixed model with repeated measures. Specific *p* values are shown when there is a significant genotype‐load interaction (*p* < 0.05).
**Supplemental Table S1.** Gauge‐site stiffness measured by gauge‐based experiment and finite element modeling
**Supplemental Table S2.** Gauge‐site strains (μeμε) determined by gauge‐based experiment and finite element modeling with selected loads
**Supplemental Table S3.** FE‐predicted peak principal strains (μeμε) in the tibial cortical and cancellous VOIs
**Supplemental Table S4.** Genes and the corresponding primer sequences for the RT‐qPCR test
**Supplemental Table S5.** CT values of GapDH and β‐Actin in femoral cortical and L_3_ to L_5_ vertebral cancellous bone for 12‐week‐old (young) and 30‐week‐old (adult) male and female ERβ‐dOT (KO) and LC mice
**Supplemental Table S6.** Fold‐change of gene expressions in femoral cortical bone for 12‐week‐old (young) and 30‐week‐old (adult) male and female ERβ‐dOT (KO) and LC mice
**Supplemental Table S7.** Serum sex steroids in 12‐week‐old and 30‐week‐old male (*A*) and female (*B*) ERβ‐dOT (KO) and LC mice
**Supplemental Table S8.** Body mass of the male and female ERβ‐dOT (KO) and LC mice before and after the loading study
**Supplemental Table S9.** Tibial length of the male and female ERβ‐dOT (KO) and LC mice at 12 weeks and 30 weeks of age
**Supplemental Table S10.** Bone morphology of L_4_ in male and female LC and ERβ‐dOT (KO) mice at 12 weeks and 30 weeks of age
**Supplemental Table S11.** Tibial morphology in male and female ERβ‐dOT (KO) and LC mice at 12 weeks and 30 weeks of age
**Supplemental Table S12.** Tibial cortical and cancellous adaptive response to compressive loading in male ERβ‐dOT (MKO) and LC (MLC) mice at 12 weeks (*A*) and 30 weeks (*B*) of age
**Supplemental Table S13.** Tibial cortical and cancellous adaptive response to compressive loading in female ERβ‐dOT (FKO) and LC (FLC) mice at 12 weeks (*A*) and 30 weeks (*B*) of age
**Supplemental Table S14.** Fold‐change of the expressions of genes in lumbar vertebrae (L_3_ to L_5_) for 12‐week‐old (young) and 30‐week‐old (adult) male ERβ‐dOT and LC mice.

## Data Availability

The data that support the findings of this study are available from the corresponding author upon reasonable request
