# Supplementary material for: Osteocyte Estrogen Receptor β (Ot‐ERβ) Regulates Bone Turnover and Skeletal Adaptive Response to Mechanical Loading Differently in Male and Female Growing and Adult Mice
Source: J Bone Miner Res. 2022 Dec 19;38(1):186–97. doi: 10.1002/jbmr.4731 (PMC10108310; doi:10.1002/jbmr.4731)
Supplement: Supplementary file 1 — Supplemental Fig. S1. Tibial cancellous adaptive response to the compressive loading in 12‐week‐old (young) and 30‐week‐old (adult) male and female LC and ERβ‐dOT (KO) mice. The structural adaptive response in cancellous bone was determined in the loaded (Loaded, red) and controlled (Control, blue) tibias of male and female LC (MLC, FLC) and ERβ‐dOT (MKO, FKO) mice at 12 weeks (A, B) and 30 weeks of age (C, D) by micro‐CT analysis. Trabecular bone volume fraction (BV/TV) of the proximal tibia is shown. Data are presented as box plots with median and interquartile ranges (IQR; 25th to 75th percentile) including all data points (n = 10–12 per group). The effects of genotype and load and their interaction were tested by the linear mixed model with repeated measures followed by pairwise comparisons with Bonferroni correction. *p < 0.05 for significant loading effect for the same genotype by the linear mixed model with repeated measures. Specific p values are shown when there is a significant genotype‐load interaction (p < 0.05). Supplemental Table S1. Gauge‐site stiffness measured by gauge‐based experiment and finite element modeling Supplemental Table S2. Gauge‐site strains (μeμε) determined by gauge‐based experiment and finite element modeling with selected loads Supplemental Table S3. FE‐predicted peak principal strains (μeμε) in the tibial cortical and cancellous VOIs Supplemental Table S4. Genes and the corresponding primer sequences for the RT‐qPCR test Supplemental Table S5. CT values of GapDH and β‐Actin in femoral cortical and L3 to L5 vertebral cancellous bone for 12‐week‐old (young) and 30‐week‐old (adult) male and female ERβ‐dOT (KO) and LC mice Supplemental Table S6. Fold‐change of gene expressions in femoral cortical bone for 12‐week‐old (young) and 30‐week‐old (adult) male and female ERβ‐dOT (KO) and LC mice Supplemental Table S7. Serum sex steroids in 12‐week‐old and 30‐week‐old male (A) and female (B) ERβ‐dOT (KO) and LC mice Supplemental Table S8. Body [file JBMR-38-186-s001.doc]

**Supplementary Table S1.** Gauge-site stiffness measured by gauge-based experiment and finite element modeling

| **10wk** |  | **MALE** | | p-value (KO vs. LC) |  | **FEMALE** | | p-value (ERβ-dOT vs. LC) |
| --- | --- | --- | --- | --- | --- | --- | --- | --- |
|  |  | MLC | MKO |  |  | FLC | FKO |  |
| **Exp stiffness** |  | 0.0082±0.0028 | 0.0088±0.0032 | 0.8 |  | 0.0056±0.0008 | 0.0054±0.0005 | 0.8 |
| **FE stiffness** |  | 0.0079±0.0005 | 0.0080±0.0021 | 1.0 |  | 0.0061±0.0008 | 0.00562±0.0008 | 0.5 |
| **p-value (Exp vs. FE)** |  | 0.5 | 0.7 |  |  | 0.4 | 0.8 |  |
| **28wk** |  | **MALE** | | p-value (KO vs. LC) |  | **FEMALE** | | p-value (ERβ-dOT vs. LC) |
|  |  | MLC | MKO |  |  | FLC | FKO |  |
| **Exp stiffness** |  | 0.0073±0.002 | 0.0063±0.0013 | 0.5 |  | 0.0063±0.0006 | 0.0059±0.0004 | 0.4 |
| **FE stiffness** |  | 0.0070±0.002 | 0.0061±0.0008 | 0.5 |  | 0.0070±0.0024 | 0.0057±0.0010 | 0.9 |
| **p-value (Exp vs. FE)** |  | 0.8 | 0.8 |  |  | 0.9 | 0.9 |  |

Data are presented as Mean ± SD (n = 3).

Abbreviation: MKO/FKO, male/female ERβ-dOT mice; MLC/FLC, male/female LC mice; Exp, experimental measurement of the stiffness at the gauge site; FE, finite element-predicted stiffness at the gauge site. Significant difference between genotypes was determined by Student’s *t*-test (p < 0.05). NS: no significant difference (p > 0.1).

**Supplementary Table S2**. Gauge-site strains (μe) determined by gauge-based experiment and finite element modeling with selected loads

| **10wk** | **MALE (-13N)** | |  | **FEMALE (-9N)** | |  | **2-way ANOVA (p-value)** | | |
| --- | --- | --- | --- | --- | --- | --- | --- | --- | --- |
|  | MLC | MKO |  | FLC | FKO |  | Genotype | Sex | Interaction |
| **Exp strain** | 1596±56 | 1613±541 |  | 1640±252 | 1664±152 |  | 0.8 | 0.7 | 1.0 |
| **FE strain** | 1648±102 | 1716±399 |  | 1481±200 | 1628±262 |  | 0.7 | 0.6 | 0.9 |
| **p-value (Exp vs. FE)** | 0.5 | 0.8 |  | 0.4 | 0.9 |  |  |  |  |
| **28wk** | **MALE (-12.5N)** | |  | **FEMALE (-11N)** | |  | **2-way ANOVA (p-value)** | | |
|  | MLC | MKO |  | FLC | FKO |  | Genotype | Sex | Interaction |
| **Exp strain** | 1813±542 | 2019±361 |  | 1771±163 | 1886±114 |  | 0.8 | 0.9 | 0.9 |
| **FE strain** | 1899±541 | 2063±246 |  | 1713±661 | 1960±307 |  | 0.8 | 0.7 | 0.9 |
| **p-value (Exp vs. FE)** | 0.9 | 0.9 |  | 0.9 | 0.7 |  |  |  |  |

Data are presented as Mean ± SD (n = 3).

Abbreviation: MKO/FKO, male/female ERβ-dOT mice; MLC/FLC, male/female LC mice; Exp, experimental measurement based on the stain at the gauge site; FE, finite element-predicted strain at the gauge site. Significant difference between Exp- and FE-measured strains at the gauge site was determined by paired *t*-test (p < 0.05).

Significant difference in Exp strain or FE strain between genotypes, sexes, and their interactions were determined by two-way ANOVA (p < 0.05).

**Supplementary Table S3.** FE-predicted peak principal strains (μe) in the tibial cortical and cancellous VOIs

| **10wk** |  | **MALE (-13N)** | |  | **FEMALE (-9N)** | |  | **2-way ANOVA (p-value)** | | |
| --- | --- | --- | --- | --- | --- | --- | --- | --- | --- | --- |
|  |  | MLC | MKO |  | FLC | FKO |  | Genotype | Sex | Interaction |
| **CANCELLOUS** |  |  |  |  |  |  |  |  |  |  |
| Peak Tens |  | 1918±583 | 2051±245 |  | 1686±83 | 1860±129 |  | 0.7 | 0.02 | 0.8 |
| Peak Comp |  | -3655±1011 | -3791±170 |  | -3211±270 | -3262±208 |  | 0.8 | 0.02 | 0.9 |
| **CORTICAL 37%** |  |  |  |  |  |  |  |  |  |  |
| Peak Tens |  | 2106±351 | 2243±441 |  | 1882±198 | 1953±86 |  | 0.9 | 0.008 | 0.8 |
| Peak Comp |  | -4089±740 | -4272±747 |  | -3959±121 | -4111±55 |  | 1.0 | 0.002 | 0.8 |
| **CORTICAL 50%** |  |  |  |  |  |  |  |  |  |  |
| Peak Tens |  | 2707±467 | 2735±460 |  | 2563±187 | 2497±400 |  | 0.8 | 0.2 | 0.9 |
| Peak Comp |  | -3696±479 | -3675±607 |  | -3338±145 | -3423±308 |  | 0.8 | 0.01 | 1.0 |
| **28wk** |  | **MALE (-12.5N)** | |  | **FEMALE (-11N)** | |  | **2-way ANOVA (p-value)** | | |
|  |  | MLC | MKO |  | FLC | FKO |  | Genotype | Sex | Interaction |
| **CANCELLOUS** |  |  |  |  |  |  |  |  |  |  |
| Peak Tens |  | 4132±491 | 4053±1024 |  | 2360±594 | 3381±512 |  | 0.9 | 0.8 | 0.2 |
| Peak Comp |  | -6345±1392 | -6702±1559 |  | -3900±1727 | -4941±379 |  | 0.8 | 0.3 | 0.6 |
| **CORTICAL 37%** |  |  |  |  |  |  |  |  |  |  |
| Peak Tens |  | 2842±754 | 3036±217 |  | 2642±729 | 2573±319 |  | 0.7 | 0.8 | 0.7 |
| Peak Comp |  | -4646±1325 | -4994±485 |  | -4306±1199 | -4022±521 |  | 0.7 | 0.4 | 0.6 |
| **CORTICAL 50%** |  |  |  |  |  |  |  |  |  |  |
| Peak Tens |  | 2900±1199 | 3115±373 |  | 2917±955 | 2867±315 |  | 0.8 | 0.7 | 0.8 |
| Peak Comp |  | -3806±1247 | -4059±476 |  | -3782±1071 | -3677±260 |  | 0.8 | 0.7 | 0.7 |

Data are presented as Mean ± SD (n = 3).

The peak compressive and tensile strains are the 95^th^ percentile minimum and maximum principal strains, respectively, which indicate the cutoff strain values within the top 5% for each VOI. Abbreviation: MKO/FKO, male/female ERβ-dOT mice; MLC/FLC, male/female LC mice; Comp, compressive strain; Tens, tensile strain. Significant difference in principal strains between genotypes was tested by Student’s *t*-test (p < 0.05). The effects of genotype and sex on the principal strains were determined by two-way ANOVA (p < 0.05).

**Supplementary Table S4.** Genes and the corresponding primer sequences for the RT-qPCR test

| **Gene** | **Protein** | **Primer Sequence (5'-3')** |
| --- | --- | --- |
| *Esr2* | ERβ | 5'-AGTCCGCCTGGAAAGC-3' 5'-AACGTGCCACTGAGGGTCTT-3' |
| *Esr1* | ERα | 5'-CCTCCCGCCTTCTACAGGT-3' 5'-CACACGGCACAGTAGCGAG-3' |
| *Ar* | AR | 5'-ATCCACACGCCCGTATCAAG-3' 5'-AAGTCCCCATAGCGGCATTG-3' |
| *Cyp19a1* | Aromatase | 5'-ATGTTCTTGGAAATGCTGAACCC-3' 5'-AGGACCTGGTATTGAAGACGAG-3' |
| *Ctsk* | Cathepsin K | 5'-GAAGAAGACTCACCAGAAGCAG-3' 5'-TCCAGGTTATGGGCAGAGATT-3' |
| *Tnfsf11* | RANKL | 5'-CAGCATCGCTCTGTTCCTGTA-3' 5'-CTGCGTTTTCATGGAGTCTCA-3' |
| *Tnfsf11b* | OPG | 5'-ACCCAGAAACTGGTCATCAGC-3' 5'-CTGCAATACACACACTCATCACT-3' |
| *Col1a1* | Type-I Collagen | 5'-CTTGGCTGTAGGGGGCATAC-3' 5'-TCTTGCGCTATTTGGCCTGA-3' |
| *Csf1* | M-CSF | 5'-GCCAGAGGGGTTTCTGTCG-3' 5'-GTTCGTGCCGCTAAAAGTCA-3' |
| *Sost* | Sclerostin | 5'-AGCCTTCAGGAATGATGCCAC-3' 5'-CTTTGGCGTCATAGGGATGGT-3' |
| *Gapdh* | GapDH | 5'-AGGTCGGTGTGAACGGATTTG-3' 5'-TGTAGACCATGTAGTTGAGGTCA-3' |
| *Actb* | β-Actin | 5'-GGCTGTATTCCCCTCCATCG-3' 5'-CCAGTTGGTAACAATGCCATGT-3' |

**Supplementary Table S5.** CT values of GapDH and β-Actin in femoral cortical and L3-5 vertebral cancellous bone for 12wk (young) and 30wk (adult) old male and female ERβ-dOT (KO) and LC mice.

| **Femoral cortical bone (no marrow)** |  | **12wk** | | **p-value (KO vs. LC)** |  | **30wk** | | **p-value (KO vs. LC)** |
| --- | --- | --- | --- | --- | --- | --- | --- | --- |
|  |  | MLC | MKO |  |  | MLC | MKO |  |
| GapDH (G*apdh*) |  | 22.4±0.9 | 22.6±0.6 | 0.9 |  | 22.1±3.8 | 19.8±1.6 | 0.04 |
| β-Actin (*Actb*) |  | 21.3±0.9 | 19.8±1.0 | 0.03 |  | 17.8±2.0 | 18.5±2.7 | 0.5 |
| **Femoral cortical bone (no marrow)** |  | **12wk** | | **p-value (KO vs. LC)** |  | **30wk** | | **p-value (KO vs. LC)** |
|  |  | FLC | FKO |  |  | FLC | FKO |  |
| GapDH (G*apdh*) |  | 22.8±0.7 | 23.1±0.5 | 0.9 |  | 21.6±1.1 | 23.1±0.5 | 0.07 |
| β-Actin (*Actb*) |  | 20.0±2.0 | 20.7±1.6 | 0.5 |  | 21.4±2.5 | 21.5±2.1 | 0.9 |
| **L3-5 Cancellous  (with marrow)** |  | **12wk** | | **p-value (KO vs. LC)** |  | **30wk** | | **p-value (KO vs. LC)** |
|  |  | MLC | MKO |  |  | MLC | MKO |  |
| GapDH (G*apdh*) |  | 24.0±0.7 | 22.3±0.8 | 0.1 |  | 22.9±1.6 | 23.1±0.6 | 0.3 |
| β-Actin (*Actb*) |  | 24.5±0.4 | 22.0±0.8 | 0.01 |  | 22.6±1.9 | 23.4±1.8 | 0.2 |
| **L3-5 Cancellous  (with marrow)** |  | **12wk** | | **p-value (KO vs. LC)** |  | **30wk** | | **p-value (KO vs. LC)** |
|  |  | FLC | FKO |  |  | FLC | FKO |  |
| GapDH (G*apdh*) |  | NA | NA |  |  | NA | NA |  |
| β-Actin (*Actb*) |  | NA | NA |  |  | NA | NA |  |

Data are presented as Mean ± SD (n = 7-10).

Total RNA was extracted from femoral cortical bone (no marrow) or L3-5 vertebral cancellous bone (with marrow) of male and female ERβ-dOT (MKO/FKO) and LC (MLC/FLC) mice at 12 wks and 30 wks of age. CT values of GapDH (G*apdh*) and β-Actin (*Actb*) tested in the RT-qPCR are shown with their gene names listed in parentheses. Significant difference in CT values between genotypes was tested by Student’s *t*-test (p < 0.05). Specific p-values are shown in the table. NA: no measurement.

**Supplementary Table S6**. Fold-change of gene expressions in femoral cortical bone for 12wk (young) and 30wk (adult) old male and female ERβ-dOT (KO) and LC mice.

| **Fold-change Femoral cortical bone** |  | **12wk** | | **p-value (KO vs. LC)** |  | **30wk** | | **p-value (KO vs. LC)** |
| --- | --- | --- | --- | --- | --- | --- | --- | --- |
|  |  | MLC | MKO |  |  | MLC | MKO |  |
| ERβ (*Esr2*) |  | 1.0±0.6 | 0.2±0.1 | 0.01 |  | 1.0±0.8 | 0.3±0.6 | 0.03 |
| ERα (*Esr1*) |  | 1.0±0.6 | 2.0±1.3 | 0.1 |  | 1.0±0.8 | 1.4±1.3 | 0.5 |
| AR (*Ar*) |  | 1.0±0.9 | 3.2±2.9 | 0.04 |  | 1.0±0.6 | 1.5±1.0 | 0.3 |
| Aromatase (*Cyp19a1*) |  | 1.0±0.8 | 1.1±1.1 | 0.8 |  | 1.0±0.9 | 1.2±1.1 | 0.9 |
| Cat K (*Ctsk*) |  | 1.0±0.9 | 3.3±1.4 | 0.004 |  | 1.0±0.5 | 1.7±0.9 | 0.03 |
| Type-I collagen (*Col1a1*) |  | 1.0±1.2 | 3.7±1.4 | 0.01 |  | 1.0±0.7 | 1.4±0.6 | 0.2 |
| RANKL (*Tnfsf11*) |  | 1.0±0.3 | 4.4±3.7 | 0.02 |  | 1.0±0.9 | 1.6±1.9 | 0.9 |
| OPG (*Tnfsf11b*) |  | 1.0±1.9 | 2.5±4.4 | 0.07 |  | 1.0±0.9 | 0.4±0.3 | 0.04 |
| RANKL/OPG |  | 1.0±0.1 | 1.1±0.6 | 0.08 |  | 1.0±0.1 | 1.1±0.1 | 0.02 |
| M-CSF (*Csf1*) |  | 1.0±1.0 | 5.4±4.4 | 0.02 |  | 1.0±0.9 | 0.9±0.6 | 0.3 |
| Sclerostin (*Sost*) |  | 1.0±0.9 | 0.8±1.1 | 0.2 |  | NA | NA |  |
| **Fold-change Femoral cortical bone** |  | **12wk** | | **p-value (KO vs. LC)** |  | **30wk** | | **p-value (KO vs. LC)** |
|  |  | FLC | FKO |  |  | FLC | FKO |  |
| ERβ (*Esr2*) |  | 1.0±1.4 | 0.02±0.0 | 0.03 |  | 1.0±0.7 | 0.1±0.1 | 0.03 |
| ERα (*Esr1*) |  | 1.0±1.4 | 1.5±1.8 | 0.4 |  | 1.0±0.7 | 1.2±0.6 | 0.3 |
| AR (*Ar*) |  | 1.0±0.8 | 1.3±1.4 | 0.5 |  | 1.0±1.1 | 1.0±0.5 | 0.7 |
| Aromatase (*Cyp19a1*) |  | 1.0±0.6 | 4.0±3.6 | 0.3 |  | 1.0±1.4 | 0.4±0.2 | 0.9 |
| Cat K (*Ctsk*) |  | 1.0±1.0 | 2.1±2.7 | 0.6 |  | 1.0±0.7 | 1.3±0.6 | 0.6 |
| Type-I collagen (*Col1a1*) |  | 1.0±1.4 | 1.8±2.1 | 0.5 |  | 1.0±1.3 | 1.3±0.8 | 0.4 |
| RANKL (*Tnfsf11*) |  | NA | NA |  |  | NA | NA |  |
| OPG (*Tnfsf11b*) |  | NA | NA |  |  | NA | NA |  |
| RANKL/OPG |  | NA | NA |  |  | NA | NA |  |
| M-CSF (*Csf1*) |  | NA | NA |  |  | NA | NA |  |
| Sclerostin (*Sost*) |  | NA | NA |  |  | NA | NA |  |

Data are presented as Mean ± SD (n = 4-10).

Total RNA was extracted from femoral cortical bone (no marrow) of male and female ERβ-dOT (MKO/FKO) and LC (MLC/FLC) mice at 12 wks and 30 wks of age. Markers tested in the RT-qPCR are shown with their gene names in parentheses. Fold-change (FC) gene expression for KO (MKO/FKO) relative to LC (MLC/FLC) (average: 1.0) was calculated using the 2^-△△Ct^ method. The significant difference in gene expressions between genotypes was tested at the delta CT level, which was calculated as CT _test gene_ – CT _housekeeping_, by Student’s *t*-test (p < 0.05). Specific p-values are shown in the table. NA: no measurement.

**Supplementary Table S7**. Serum sex steroids in 12wk and 30wk old male (A) and female (B) ERβ-dOT (KO) and LC mice.

| **A. MALE** |  | **12wk** | | **p-value (KO vs. LC)** |  | **30wk** | | **p-value (KO vs. LC)** |
| --- | --- | --- | --- | --- | --- | --- | --- | --- |
|  |  | MLC | MKO |  |  | MLC | MKO |  |
| Estradiol (E2), pg/ml |  | 0 | 0 | > 0.9 |  | 0 | 0 | > 0.9 |
| Estrone (E1), pg/ml |  | 0 | 0 | > 0.9 |  | 0 | 0 | > 0.9 |
| Testosterone (T), pg/ml |  | 533.0±421.6 | 5343.4±9343.8 | 0.9 |  | 5430.4±8546.1 | 5742.5±6111.5 | 0.5 |
| Dihydrotestosterone (DHT), pg/ml |  | 33.4±16.4 | 176.6±267.0 | 0.5 |  | 164.4±230.9 | 187.4±234.3 | 0.5 |
| Androstenedione (AE), pg/ml |  | 36.1±20.6 | 287.6±325.4 | 0.1 |  | 256.3±272.1 | 244.6±199.5 | > 0.9 |
| Progesterone (P), pg/ml |  | 1530.8±688.6 | 1625.9±332.0 | 0.5 |  | 1007.2±367.1 | 1134.7±418.2 | 0.6 |
| **B. FEMALE** |  | **12wk** | | **p-value (KO vs. LC)** |  | **30wk** | | **p-value (KO vs. LC)** |
|  |  | FLC | FKO |  |  | FLC | FKO |  |
| Estradiol (E2), pg/ml |  | 1.6±2.4 | 4.4±5.6 | 0.1 |  | 2.0±5.0 | 7.6±9.4 | 0.06 |
| Estrone (E1), pg/ml |  | 0.0±0.0 | 0.4±0.8 | > 0.9 |  | 0.8±1.3 | 0.8±1.1 | 0.7 |
| Testosterone (T), pg/ml |  | 25.9±9.4 | 34.8±16.2 | 0.3 |  | 39.5±15.7 | 56.4±45.2 | 0.7 |
| Dihydrotestosterone (DHT), pg/ml |  | 2.4±2.0 | 2.4±1.8 | 1.0 |  | 9.5±9.4 | 10.4±9.9 | 0.9 |
| Androstenedione (AE), pg/ml |  | 30.7±7.4 | 74.3±27.9 | 0.06 |  | 39.9±15.7 | 28.9±4.1 | 0.4 |
| Progesterone (P), pg/ml |  | 1670.5±772.6 | 3283.2±1977.9 | 0.07 |  | 3543.9±3402.6 | 3849.2±6426.2 | 0.2 |

Data are presented as Mean ± SD (n = 7-10).

Blood serum was collected from male and female ERβ-dOT (MKO/FKO) and LC (MLC/FLC) mice at 12 wks and 30 wks of age. Six sex steroids tested by the GC-MS/MS are shown in the table with their abbreviations listed in parentheses. The significant difference in sex steroid concentrations between genotypes was tested using the Mann-Whitney test (p < 0.05). The levels of E1 and E2 in 12wk and 30wk old MLC and MKO were below the ‘Lower Limit of Quantification (LLOQ, 0.5 pg/ml for E1 and 0.75 pg/ml for E2) and reported as ‘0’.^(47)^

**Supplementary Table S8**. Body mass of the male and female ERβ-dOT (KO) and LC mice before and after the loading study

| **Body Mass (g)** | **MALE** | | **p-value (LC vs. KO)** |  | **FEMALE** | | **p-value (LC vs. KO)** |
| --- | --- | --- | --- | --- | --- | --- | --- |
|  | MLC | MKO |  |  | FLC | FKO |  |
| 10wk | 25.1±1.5 | 26.8±1.7 | 0.1 |  | 20.7±1.6 | 20.6±1.5 | 0.8 |
| 12wk | 25.1±1.8 | 26.7±1.7 | 0.2 |  | 20.4±1.1 | 20.4±1.6 | 0.6 |
| Difference (10 vs. 12wk) | 0.09% | -0.1% |  |  | -1.3% | -0.9% |  |
| p-value (10 vs. 12 wk) | 0.9 | 0.9 |  |  | 0.9 | 1.0 |  |
| **Body Mass (g)** | **MALE** | | **p-value (LC vs. KO)** |  | **FEMALE** | | **p-value (LC vs. KO)** |
|  | MLC | MKO |  |  | FLC | FKO |  |
| 28wk | 32.8±2.8 | 31.4±2.0 | 0.2 |  | 23.5±6.9 | 25.7±2.4 | 0.1 |
| 30wk | 30.8±2.2 | 29.6±1.9 | 0.2 |  | 22.6±6.6 | 24.3±2.0 | 0.2 |
| Difference (28 vs. 30wk) | -5.8% | -5.8% |  |  | -3.2% | -5.4% |  |
| p-value (28 vs. 30 wk) | 0.9 | 0.07 |  |  | 0.7 | 0.9 |  |

Data are presented as Mean ± SD (n = 10-12)

Abbreviation: MKO/FKO, male/female ERβ-dOT mice; MLC/FLC, male/female LC mice. Difference: Percent difference (%) between ERβ-dOT and LC calculated as 100*(ERβ-dOT - LC)/LC. Significant difference in body mass between genotypes and ages was by Student’s *t*-test is indicated at p < 0.05.

**Supplementary Table S9**. Tibial length of the male and female ERβ-dOT (KO) and LC mice at 12 wks and 30 wks of age.

| **12wk Tibial Length (mm)** | **MALE** | | **p-value (Loaded vs. Control)** |  | **FEMALE** | | **p-value (Loaded vs. Control)** |
| --- | --- | --- | --- | --- | --- | --- | --- |
|  | Control | Loaded |  |  | Control | Loaded |  |
| LC | 17.9±0.2 | 18.1±0.6 | 0.2 |  | 17.5±0.2 | 17.5±0.2 | 0.3 |
| KO | 18±0.3 | 17.9±0.2 | 0.2 |  | 17.4±0.3 | 17.5±0.4 | 0.4 |
| p-value (LC vs. KO) | 0.5 | 0.4 |  |  | 0.8 | 0.8 |  |
| **30wk Tibial Length (mm)** | **MALE** | | **p-value (Loaded vs. Control)** |  | **FEMALE** | | **p-value (Loaded vs. Control)** |
|  | Control | Loaded |  |  | Control | Loaded |  |
| LC | 18.5±0.2 | 18.5±0.2 | 0.4 |  | 18.3±0.1 | 18.3±0.2 | 0.5 |
| KO | 18.4±0.2 | 18.4±0.1 | 0.4 |  | 18.4±0.2 | 18.3±0.1 | 0.1 |
| p-value (LC vs. KO) | 0.1 | 0.2 |  |  | 0.3 | 0.3 |  |

Data are presented as Mean ± SD (n = 10-12)

Abbreviation: MKO/FKO, male/female ERβ-dOT mice; MLC/FLC, male/female LC mice. Significant difference in tibial length between genotypes or loaded and control limbs was tested by Student’s *t*-test (p < 0.05).

**Supplementary Table S10.** Bone morphology of L4 in male and female LC and ERβ-dOT (KO) mice at 12 wks and 30 wks of age

| **12wk  L4 Body** |  | **MALE** | | Diff (%) |  | **FEMALE** | | Diff (%) |
| --- | --- | --- | --- | --- | --- | --- | --- | --- |
|  |  | MLC | MKO |  |  | FLC | FKO |  |
| **CANCELLOUS** |  | n = 10 | n = 10 |  |  | n = 10 | n = 10 |  |
| BV/TV, % |  | 45.9±1.5 | 47.4±1.1 ^a^ | 3.10 |  | 40.3±0.8 | 39.4±2.0 | -1.6 |
| Tb. Th, mm |  | 0.113±0.003 | 0.114±0.009 | 0.80 |  | 0.094±0.004 | 0.092±0.005 | -2.3 |
| Tb. Sp, μm |  | 1.09±0.07 | 1.03±0.05 ^a^ | -5.90 |  | 1.32±0.07 | 1.45±0.01 ^a^ | 7.7 |
| Tb. N, 1/mm |  | 71.8±3.4 | 77.3±5.6 ^a^ | 7.60 |  | 71.9±3.7 | 71.5±6.2 | 7.9 |
| Tb. BV, mm^3^ |  | 1.57±0.13 | 1.75±0.17 ^a^ | 11.70 |  | 1.31±0.09 | 1.28±0.09 | -2.4 |
| Tb. TV, mm^3^ |  | 3.41±0.22 | 3.67±0.33 ^a^ | 8.30 |  | 3.27±0.15 | 3.25±0.152 | -0.8 |
| Tb. BS, mm^2^ |  | 59.1±3.2 | 63.9±4.1 ^a^ | 8.20 |  | 58.2±3.4 | 58.2±4.6 | 0.0 |
| **CORTICAL** |  |  |  |  |  |  |  |  |
| Ct. Ar, mm^2^ |  | 0.43±0.023 | 0.44±0.02 | 0.90 |  | 0.36±0.01 | 0.34±0.02 | -7.3 |
| Ct. BV, mm^3^ |  | 1.41±0.09 | 1.43±0.07 | 1.20 |  | 1.16±0.05 | 1.11±0.06 | -7.0 |
| Ct. Th, mm |  | 0.065±0.003 | 0.064±0.002 | -1.70 |  | 0.054±0.004 | 0.052±0.004 | -3.7 |
| **30wk  L4 Body** |  | **MALE** | | Diff (%) |  | **FEMALE** | | Diff (%) |
|  |  | MLC | MKO |  |  | FLC | FKO |  |
| **CANCELLOUS** |  | n = 12 | n = 11 |  |  | n = 10 | n = 11 |  |
| BV/TV, % |  | 43.5±2.1 | 41.3±2.1 ^a^ | -5.00 |  | 37.4±2.7 | 37.8±3.4 | 1.1 |
| Tb. Th, mm |  | 0.114±0.004 | 0.109±0.004 ^a^ | -3.5 |  | 0.107±0.006 | 0.106±0.003 | -0.7 |
| Tb. Sp, μm |  | 1.27±0.07 | 1.33±0.12 | 4 |  | 1.73±0.2 | 1.73±0.34 | 0.3 |
| Tb. N, 1/mm |  | 79.3±3.4 | 74.3±5.3 ^a^ | -6.30 |  | 63.4±5.3 | 63.4±4.8 | 0.0 |
| Tb. BV, mm^3^ |  | 1.80±0.15 | 1.65±0.18 ^a^ | -8.6 |  | 1.27±0.18 | 1.34±0.20 | 5.1 |
| Tb. TV, mm^3^ |  | 4.16±0.20 | 3.93±0.28 ^a^ | -5.50 |  | 3.42±0.33 | 3.55±0.47 | 3.8 |
| Tb. BS, mm^2^ |  | 67.1±3.6 | 62.7±5.4 ^a^ | -6.50 |  | 54.6±4.3 | 55.5±5.3 | 1.6 |
| **CORTICAL** |  |  |  |  |  |  |  |  |
| Ct. Ar, mm^2^ |  | 0.41±0.02 | 0.41±0.01 | -1.90 |  | 0.41±0.03 | 0.41±0.01 | -0.4 |
| Ct. BV, mm^3^ |  | 1.54±0.11 | 1.44±0.06 ^a^ | -6.50 |  | 1.45±0.11 | 1.44±0.02 | -0.2 |
| Ct. Th, mm |  | 0.062±0.003 | 0.063±0.003 | 1.30 |  | 0.060±0.003 | 0.060±0.002 | -0.2 |

Data are presented as Means ± SD (n = 10-12).
Abbreviation: MKO/FKO, male/female ERβ-dOT mice; MLC/FLC, male/female LC mice. BV/TV, trabecular bone volume fraction; Tb. Th, trabecular thickness; Tb. Sp, trabecular separation; Tb. N, trabecular number; Tb. BV, trabecular bone volume; Tb. TV, tissue volume of trabecular bone; Tb. BS: trabecular bone surface; Ct. Ar, cortical bone area; Ct. BV, cortical bone volume; Ct. Th, cortical thickness. ^a^ *p* < 0.05 for significant difference between genotypes by Student’s *t*-test. Diff: Percent difference (%) between ERβ-dOT (KO) and LC calculated as 100*(KO – LC)/LC.

**Supplementary Table S11.** Tibial morphology in male and female ERβ-dOT (KO) and LC mice at 12 wks and 30 wks of age

| **12wk**  **Tibia** |  | **MALE** | | Diff (%) |  | **FEMALE** | | Diff (%) |
| --- | --- | --- | --- | --- | --- | --- | --- | --- |
|  |  | **LC** | **KO** |  |  | **LC** | **KO** |  |
| **CANCELLOUS** |  | n = 10 | n = 10 |  |  | n = 10 | n = 10 |  |
| BV/TV, % |  | 28.8±6.5 | 30.7±6.3 | 6.9 |  | 15.8±1.2 | 15.8±0.1 | -0.1 |
| Tb. N, 1/mm |  | 5.75±0.27 | 5.83±0.27 | 1.4 |  | 4.02±0.29 | 4.05±0.55 | 0.7 |
| Tb. Th, mm |  | 0.066±0.009 | 0.068±0.009 | 3.3 |  | 0.060±0.002 | 0.058±0.002 | -3.5 |
| Tb. Sp, mm |  | 0.154±0.008 | 0.151±0.10 | -2.2 |  | 0.239±0.021 | 0.242±0.039 | 1.4 |
| Tb. BV, mm^3^ |  | 0.56±0.15 | 0.63±0.13 | 13.1 |  | 0.26±0.04 | 0.25±0.03 | -4.2 |
| Tb. TV, mm^3^ |  | 2.07±0.17 | 2.20±0.14 | 6.3 |  | 1.60±0.23 | 1.72±0.05 | 7.8 |
| Tb. BMD, mg HA/mm^3^ |  | 741.6±11.5 | 742.1±7.9 | 0.1 |  | 756.3±21.3 | 734.7±33.7 | -2.9 |
| **CORTICAL, 37%** |  |  |  |  |  |  |  |  |
| Ct. Ar, mm^2^ |  | 0.912±0.126 | 0.956±0.076 | 4.8 |  | 0.718±0.011 | 0.701±0.032 | -2.3 |
| Ct. BV, mm^3^ |  | 0.391±0.053 | 0.409±0.031 | 4.7 |  | 0.308±0.004 | 0.299±0.015 | -2.8 |
| Imax, mm^4^ |  | 0.325±0.073 | 0.343±0.066 | 5.5 |  | 0.207±0.016 | 0.191±0.017 | -7.4 |
| Imin, mm^4^ |  | 0.109±0.027 | 0.116±0.021 | 7.2 |  | 0.066±0.006 | 0.062±0.007 | -5.2 |
| Ct. BMD, mg HA/mm^3^ |  | 1030.4±17.0 | 1041.1.1±13.9 | 1.0 |  | 1047.2±9.6 | 1051.9±14.7 | 0.5 |
| **CORTICAL, 50%** |  |  |  |  |  |  |  |  |
| Ct. Ar, mm^2^ |  | 0.758±0.110 | 0.793±0.074 | 4.5 |  | 0.568±0.023 | 0.553±0.021 | -2.6 |
| Ct. BV, mm^3^ |  | 0.324±0.047 | 0.339±0.030 | 4.7 |  | 0.243±0.011 | 0.236±0.010 | -3.0 |
| Imax, mm^4^ |  | 0.121±0.031 | 0.129±0.023 | 6.9 |  | 0.065±0.005 | 0.060±0.005 | -6.4 |
| Imin, mm^4^ |  | 0.082±0.021 | 0.089±0.016 | 8.6 |  | 0.053±0.004 | 0.049±0.002 | -6.4 |
| Ct. BMD, mg HA/mm^3^ |  | 1119.1±22.1 | 1129.8±17.1 | 1.0 |  | 1106.0±15.7 | 1043.4±21.8 ^a^ | -5.7 |
| **30wk**  **Tibia** |  | **MALE** | | Diff (%) |  | **FEMALE** | | Diff (%) |
|  |  | **LC** | **KO** |  |  | **LC** | **KO** |  |
| **CANCELLOUS** |  | n = 12 | n = 11 |  |  | n = 10 | n = 11 |  |
| BV/TV, % |  | 15.2±3.2 | 15.4±2.7 | 1.3 |  | 7.4±1.8 | 7.0±0.8 | -0.2 |
| Tb. N, 1/mm |  | 4.07±0.25 | 4.09±0.24 | 0.5 |  | 2.53±0.08 | 2.60±0.24 | 2.9 |
| Tb. Th, mm |  | 0.059±0.003 | 0.058±0.002 | -1.2 |  | 0.067±0.005 | 0.065±0.003 | -2.0 |
| Tb. Sp, mm |  | 0.230±0.017 | 0.229±0.014 | -0.3 |  | 0.400±0.028 | 0.394±0.038 | -0.9 |
| Tb. BV, mm^3^ |  | 0.31±0.09 | 0.32±0.06 | 3.2 |  | 0.14±0.02 | 0.16±0.11 | 12.6 |
| Tb. TV, mm^3^ |  | 2.06±0.15 | 2.11±0.36 | 2.3 |  | 1.76±0.17 | 1.79±0.23 | 1.4 |
| Tb. BMD, mg HA/mm^3^ |  | 768.0±22.8 | 761.2±22.0 | -0.9 |  | 819.1±31.1 | 813.0±19.9 | -0.7 |
| **CORTICAL, 37%** |  |  |  |  |  |  |  |  |
| Ct. Ar, mm^2^ |  | 0.832±0.046 | 0.840±0.040 | 0.9 |  | 0.755±0.026 | 0.774±0.039 | 2.5 |
| Ct. BV, mm^3^ |  | 0.357±0.021 | 0.363±0.021 | 1.6 |  | 0.332±0.017 | 0.335±0.020 | 0.9 |
| Imax, mm^4^ |  | 0.294±0.038 | 0.291±0.031 | -1.1 |  | 0.220±0.021 | 0.216±0.028 | -1.8 |
| Imin, mm^4^ |  | 0.092±0.0140 | 0.100±0.010 | 7.3 |  | 0.068±0.004 | 0.070±0.010 | 2.3 |
| Ct. BMD, mg HA/mm^3^ |  | 1064.2±23.9 | 1082.1±15.1 | 1.7 |  | 1091.8±15.5 | 1094.5±23.6 | 0.3 |
| **CORTICAL, 50%** |  |  |  |  |  |  |  |  |
| Ct. Ar, mm^2^ |  | 0.699±0.051 | 0.683±0.037 | -2.3 |  | 0.605±0.022 | 0.610±0.034 | 0.9 |
| Ct. BV, mm^3^ |  | 0.303±0.026 | 0.299±0.024 | -1.1 |  | 0.267±0.014 | 0.266±0.018 | -0.6 |
| Imax, mm^4^ |  | 0.124±0.024 | 0.126±0.012 | 1.7 |  | 0.075±0.005 | 0.075±0.008 | 0.3 |
| Imin, mm^4^ |  | 0.076±0.009 | 0.080±0.009 | 4.2 |  | 0.060±0.004 | 0.060±0.010 | -1.1 |
| Ct. BMD, mg HA/mm^3^ |  | 1159.6±13.4 | 1156.3±14.4 | -0.3 |  | 1149.2±25.3 | 1151.0±32.7 | 0.2 |

**Supplementary Table S11**

Data are presented as Mean ± SD (n = 10-12).
Abbreviation: MKO/FKO, male/female ERβ-dOT mice; MLC/FLC, male/female LC mice. BV/TV, trabecular bone volume fraction; Tb. Th, trabecular thickness; Tb. Sp, trabecular separation; Tb. N, trabecular number; Tb. BV, trabecular bone volume; Tb. TV, total volume of trabecular bone; Tb. BMD, trabecular bone mineral density; Ct. Ar, cortical bone area; Ct. BV, cortical bone volume; Imax: maximum moments of inertia; Imin, minimum moments of inertia; Ct. BMD, cortical bone mineral density.

^a^ *p* < 0.05 for the significant difference between genotypes by Student’s *t*-test. Diff: Percent difference (%) between genotypes calculated as 100*(KO – LC)/LC.

**Supplementary Table S12.** Tibial cortical and cancellous adaptive response to compressive loading in male ERβ-dOT (MKO) and LC (MLC) mice at 12 wks (A) and 30 wks (B) of age

| **A. 12wk** | |  | **MLC** | | | Diff (%) |  | **MKO** | | | Diff (%) |  | **2-way ANOVA Results (p-value)** | | |
| --- | --- | --- | --- | --- | --- | --- | --- | --- | --- | --- | --- | --- | --- | --- | --- |
|  |  |  | **Control** | | **Loaded** |  |  | **Control** | **Loaded** | |  |  | Limb | Genotype | Interaction |
| **CANCELLOUS** | |  | n = 10 | | n = 10 |  |  | n = 10 | n = 10 | |  |  |  |  |  |
| BV/TV, % | |  | 28.8±6.5 | | 33.9±2.3 ^a^ | 17.7 |  | 30.7±6.3 | 34.9±2.9 ^a^ | | 13.3 |  | < 0.001 | 0.5 | 0.6 |
| Tb. N, 1/mm | |  | 5.75±0.27 | | 5.57±0.31 | -3.1 |  | 5.83±0.27 | 5.92±0.17 | | 1.6 |  | 0.2 | 0.1 | 0.1 |
| Tb. Th, mm | |  | 0.066±0.009 | | 0.079±0.002 ^a^ | 19.1 |  | 0.068±0.009 | 0.077±0.001 ^a^ | | 13.4 |  | 0.003 | 0.7 | 0.5 |
| Tb. Sp, mm | |  | 0.154±0.008 | | 0.150±0.010 ^a^ | -2.5 |  | 0.151±0.10 | 0.146±0.008 ^a^ | | -3.3 |  | < 0.001 | 0.3 | 0.7 |
| Tb. BV, mm^3^ | |  | 0.56±0.15 | | 0.69±0.13 ^a^ | 24.0 |  | 0.63±0.13 | 0.72±0.09 ^a^ | | 14.4 |  | < 0.001 | 0.8 | 0.8 |
| Tb. TV, mm^3^ | |  | 2.07±0.17 | | 2.06±0.16 | -0.2 |  | 2.20±0.14 | 2.13±0.12 | | -3.3 |  | 0.3 | 0.7 | 0.8 |
| Tb. BMD, mg HA/mm^3^ | |  | 741.6±11.5 | | 756.4±17.0 ^a^ | 2.0 |  | 742.1±7.9 | 754.1±13.1 ^a^ | | 1.6 |  | < 0.001 | 0.8 | 0.7 |
| **CORTICAL, 37%** | |  |  | |  |  |  |  |  | |  |  |  |  |  |
| Ct. Ar, mm^2^ | |  | 0.912±0.126 | | 1.116±0.025 ^a^ | 22.4 |  | 0.956±0.076 | 1.037±0.057 ^a,b^ | | 8.6 ^§^ |  | < 0.001 | 0.5 | 0.01 |
| Ct. BV, mm^3^ | |  | 0.391±0.053 | | 0.485±0.019 ^a^ | 24.0 |  | 0.409±0.031 | 0.450±0.027 ^a,b^ | | 10.0 ^§^ |  | < 0.001 | 0.6 | 0.01 |
| Imax, mm^4^ | |  | 0.325±0.073 | | 0.405±0.048 ^a^ | 24.6 |  | 0.343±0.066 | 0.385±0.045 ^a,b^ | | 12.2 ^§^ |  | < 0.001 | 1.0 | 0.03 |
| Imin, mm^4^ | |  | 0.109±0.027 | | 0.150±0.016 ^a^ | 38.3 |  | 0.116±0.021 | 0.140±0.016 ^a,b^ | | 19.8 ^§^ |  | < 0.001 | 0.9 | 0.03 |
| Ct. BMD, mg HA/mm^3^ | |  | 1030.4±17.0 | | 1033.6±23.5 | 0.3 |  | 1116.1±246.5 | 1044.1±14.1 | | 0.3 |  | p = 0.4 | 0.3 | 0.4 |
| **CORTICAL, 50%** | |  |  | |  |  |  |  |  | |  |  |  |  |  |
| Ct. Ar, mm^2^ | |  | 0.758±0.110 | | 0.886±0.047 ^a^ | 16.8 |  | 0.793±0.074 | 0.844±0.050 ^a^ | | 6.5 |  | < 0.001 | 1.0 | 0.1 |
| Ct. BV, mm^3^ | |  | 0.324±0.047 | | 0.373±0.016 ^a^ | 15.3 |  | 0.339±0.030 | 0.361±0.019 ^a^ | | 6.5 |  | < 0.001 | 0.8 | 0.1 |
| Imax, mm^4^ | |  | 0.121±0.031 | | 0.151±0.013 ^a^ | 25.5 |  | 0.129±0.023 | 0.143±0.017 ^a^ | | 11.0 |  | < 0.001 | 1.0 | 0.1 |
| Imin, mm^4^ | |  | 0.082±0.021 | | 0.095±0.012 ^a^ | 16.2 |  | 0.089±0.016 | 0.096±0.013 ^a^ | | 7.2 |  | < 0.001 | 0.1 | 0.1 |
| Ct. BMD, mg HA/mm^3^ | |  | 1119.1±22.1 | | 1112.6±26.9 | -0.6 |  | 1129.8±17.1 | 1125.7±19.2 | | -0.4 |  | 0.3 | 0.8 | 0.8 |
| **B. 30wk** |  | | **MLC** | | | Diff (%) |  | **MKO** | | | Diff (%) |  | **2-way ANOVA Results (p-value)** | | |
|  |  | | **Control** | **Loaded** | |  |  | **Control** | | **Loaded** |  |  | Limb | Genotype | Interaction |
| **CANCELLOUS** |  | | n = 12 | n = 12 | |  |  | n = 11 | | n = 11 |  |  |  |  |  |
| BV/TV, % |  | | 15.2±3.2 | 21.2±4.0 ^a^ | | 40.0 |  | 15.4±2.7 | | 21.9±3.7 ^a^ | 42.8 |  | < 0.001 | 0.9 | 0.9 |
| Tb. N, 1/mm |  | | 4.07±0.25 | 4.22±0.34 | | 3.7 |  | 4.09±0.24 | | 4.21±0.30 | 2.8 |  | 0.01 | 1.0 | 0.7 |
| Tb. Th, mm |  | | 0.059±0.003 | 0.079±0.004 ^a^ | | 33.4 |  | 0.058±0.002 | | 0.078±0.004 ^a^ | 32.7 |  | < 0.001 | 0.6 | 0.7 |
| Tb. Sp, mm |  | | 0.230±0.017 | 0.217±0.018 ^a^ | | -6.4 |  | 0.229±0.014 | | 0.218±0.017 ^a^ | -4.7 |  | 0.003 | 0.9 | 0.3 |
| Tb. BV, mm^3^ |  | | 0.31±0.09 | 0.45±0.14 ^a^ | | 46.28 |  | 0.32±0.06 | | 0.47±0.11 ^a^ | 28.9 |  | < 0.001 | 0.7 | 0.6 |
| Tb. TV, mm^3^ |  | | 2.06±0.15 | 2.11±0.25 | | 2.32 |  | 2.11±0.36 | | 2.01±0.07 | -4.8 |  | 0.7 | 0.6 | 0.5 |
| Tb. BMD, mg HA/mm^3^ |  | | 768.0±22.8 | 807.8±19.2 ^a^ | | 5.2 |  | 761.2±22.0 | | 803.4±15.2 ^a^ | 5.5 |  | < 0.001 | 0.4 | 0.6 |
| **CORTICAL, 37%** |  | |  |  | |  |  |  | |  |  |  |  |  |  |
| Ct. Ar, mm^2^ |  | | 0.832±0.046 | 1.063±0.038 ^a^ | | 27.8 |  | 0.840±0.040 | | 1.013±0.049 ^a,b^ | 20.7 ^§^ |  | < 0.001 | 0.1 | 0.04 |
| Ct. BV, mm^3^ |  | | 0.357±0.021 | 0.457±0.019 ^a^ | | 28.0 |  | 0.363±0.021 | | 0.437±0.017 ^a,b^ | 20.3 ^§^ |  | < 0.001 | 0.2 | 0.04 |
| Imax, mm^4^ |  | | 0.294±0.038 | 0.368±0.031 ^a^ | | 25.0 |  | 0.291±0.031 | | 0.335±0.017 ^a,b^ | 15.0 ^§^ |  | < 0.001 | 0.1 | 0.01 |
| Imin, mm^4^ |  | | 0.092±0.0140 | 0.133±0.012 ^a^ | | 44.1 |  | 0.100±0.010 | | 0.126±0.009 ^a^ | 27.3 |  | < 0.001 | 1.0 | 0.06 |
| Ct. BMD, mg HA/mm^3^ |  | | 1064.2±23.9 | 1090.0±12.2 ^a^ | | -2.4 |  | 1082.1±15.1 | | 1090.5±25.0 | 0.8 |  | 0.002 | 0.2 | 0.09 |
| **CORTICAL, 50%** |  | |  |  | |  |  |  | |  |  |  |  |  |  |
| Ct. Ar, mm^2^ |  | | 0.699±0.051 | 0.783±0.060 ^a^ | | 12.0 |  | 0.683±0.037 | | 0.777±0.021 ^a^ | 13.8 |  | < 0.001 | 0.5 | 0.6 |
| Ct. BV, mm^3^ |  | | 0.303±0.026 | 0.337±0.030 ^a^ | | 11.4 |  | 0.299±0.024 | | 0.333±0.018 ^a^ | 11.3 |  | < 0.001 | 0.7 | 0.7 |
| Imax, mm^4^ |  | | 0.124±0.024 | 0.141±0.020 ^a^ | | 13.9 |  | 0.126±0.012 | | 0.139±0.011 ^a^ | 10.0 |  | < 0.001 | 0.9 | 0.9 |
| Imin, mm^4^ |  | | 0.076±0.009 | 0.083±0.008 ^a^ | | 8.8 |  | 0.080±0.009 | | 0.085±0.007 ^a^ | 7.4 |  | < 0.001 | 0.5 | 0.5 |
| Ct. BMD, mg HA/mm^3^ |  | | 1159.6±13.4 | 1160.8±22.5 | | 0.1 |  | 1156.3±14.4 | | 1168.7±15.5 ^a^ | 1.1 |  | 0.2 | 0.7 | 0.7 |

**Supplementary Table S13.** Tibial cortical and cancellous adaptive response to compressive loading in female ERβ-dOT (FKO) and LC (FLC) mice at 12 wks (A) and 30 wks (B) of age

| **A. 12wk** |  | **FLC** | | Diff (%) | |  | | **FKO** | | Diff (%) | |  | **2-way ANOVA Results (p-value)** | | |
| --- | --- | --- | --- | --- | --- | --- | --- | --- | --- | --- | --- | --- | --- | --- | --- |
|  |  | Control | Loaded |  |  |  | | Control | Loaded |  |  |  | Limb | Genotype | Interaction |
| **CANCELLOUS** |  | n = 10 | n = 10 |  |  | | n = 10 | | n = 10 | |  |  |  |  |  |
| BV/TV, % |  | 15.8±1.2 | 18.8±1.5 ^a^ | 19.3 |  | | 15.8±0.1 | | 18.7±2.2 ^a^ | | 18.4 |  | < 0.001 | 0.8 | 0.8 |
| Tb. N, 1/mm |  | 4.02±0.29 | 4.11±0.25 | 2.2 |  | | 4.05±0.55 | | 4.22±0.37 | | 4.3 |  | 0.07 | 0.6 | 0.6 |
| Tb. Th, mm |  | 0.060±0.002 | 0.069±0.002 ^a^ | 14.9 |  | | 0.058±0.002 | | 0.068±0.004 ^a^ | | 17.4 |  | < 0.001 | 0.04 | 0.5 |
| Tb. Sp, mm |  | 0.239±0.021 | 0.232±0.017 ^a^ | -2.6 |  | | 0.242±0.039 | | 0.225±0.024 ^a^ | | -7.1 |  | 0.02 | 0.8 | 0.3 |
| Tb. BV, mm^3^ |  | 0.26±0.04 | 0.32±0.02 ^a^ | 23.4 |  | | 0.25±0.03 | | 0.32±0.02 ^a^ | | 30.0 |  | < 0.001 | 0.8 | 0.4 |
| Tb. TV, mm^3^ |  | 1.60±0.23 | 1.68±0.08 | 5.0 |  | | 1.72±0.05 | | 1.68±0.15 | | -2.3 |  | 0.7 | 0.8 | 0.5 |
| Tb. BMD, mg HA/mm^3^ |  | 756.3±21.3 | 770.4±18.1 ^a^ | 1.9 |  | | 734.7±33.7 | | 766.5±25.5 ^a^ | | 4.3 |  | < 0.001 | 0.8 | 0.5 |
| **CORTICAL, 37%** |  |  |  |  |  | |  | |  | |  |  |  |  |  |
| Ct. Ar, mm^2^ |  | 0.718±0.011 | 0.839±0.035 ^a^ | 16.9 |  | | 0.701±0.032 | | 0.872±0.019 ^a,b^ | | 24.3 ^§^ |  | < 0.001 | 0.6 | 0.004 |
| Ct. BV, mm^3^ |  | 0.308±0.004 | 0.364±0.018 ^a^ | 18.2 |  | | 0.299±0.015 | | 0.368±0.014 ^a^ | | 22.9 |  | < 0.001 | 0.7 | 0.1 |
| Imax, mm^4^ |  | 0.207±0.016 | 0.247±0.019 ^a^ | 19.3 |  | | 0.191±0.017 ^b^ | | 0.252±0.018 ^a^ | | 31.8 ^§^ |  | < 0.001 | 0.3 | 0.001 |
| Imin, mm^4^ |  | 0.066±0.006 | 0.086±0.008 ^a^ | 31.2 |  | | 0.062±0.007 | | 0.085±0.007 ^a^ | | 36.2 |  | < 0.001 | 0.4 | 0.6 |
| Ct. BMD, mg HA/mm^3^ |  | 1047.2±9.6 | 1060.6±6.8 ^a^ | 1.3 |  | | 1051.9±14.7 | | 1114.2±2.7 ^a,b^ | | 5.9 ^§^ |  | < 0.001 | < 0.001 | 0.001 |
| **CORTICAL, 50%** |  |  |  |  |  | |  | |  | |  |  |  |  |  |
| Ct. Ar, mm^2^ |  | 0.568±0.023 | 0.669±0.027 ^a^ | 17.7 |  | | 0.553±0.021 | | 0.704±0.031 ^a,b^ | | 27.2 ^§^ |  | < 0.001 | 0.3 | 0.01 |
| Ct. BV, mm^3^ |  | 0.243±0.011 | 0.286±0.011 ^a^ | 17.7 |  | | 0.236±0.010 | | 0.299±0.012 ^a,b^ | | 26.9 ^§^ |  | < 0.001 | 0.5 | 0.02 |
| Imax, mm^4^ |  | 0.065±0.005 | 0.083±0.006 ^a^ | 28.7 |  | | 0.060±0.005 | | 0.087±0.009 ^a^ | | 44.5 |  | < 0.001 | 1.0 | 0.08 |
| Imin, mm^4^ |  | 0.053±0.004 | 0.062±0.006 ^a^ | 17.4 |  | | 0.049±0.002 | | 0.062±0.006 ^a^ | | 25.7 |  | < 0.001 | 0.4 | 0.28 |
| Ct. BMD, mg HA/mm^3^ |  | 1106.0±15.7 | 1109.5±18.1 | 0.3 |  | | 1043.4±21.8 ^b^ | | 1115.8±17.8 ^a^ | | 6.9 ^§^ |  | < 0.001 | < 0.001 | < 0.001 |
| **B. 30wk** |  | **FLC** | | Diff (%) | |  | | **FKO** | | Diff (%) | |  | **2-way ANOVA Results (p-value)** | | |
|  |  | Control | Loaded |  |  |  | | Control | Loaded |  |  |  | Limb | Genotype | Interaction |
| **CANCELLOUS** |  | n = 10 | n = 10 |  |  | | n = 11 | | n = 11 | |  |  |  |  |  |
| BV/TV, % |  | 7.4±1.8 | 9.9±1.9 ^a^ | 34.3 |  | | 7.3±1.6 | | 9.7±2.6 ^a^ | | 31.8 |  | < 0.001 | 0.5 | 0.9 |
| Tb. N, 1/mm |  | 2.53±0.08 | 2.57±0.28 | 2.0 |  | | 2.60±0.24 | | 2.59±0.34 | | -0.2 |  | 0.5 | 0.5 | 0.5 |
| Tb. Th, mm |  | 0.067±0.005 | 0.084±0.009 ^a^ | 25.7 |  | | 0.065±0.003 | | 0.082±0.009 ^a^ | | 26.2 |  | < 0.001 | 0.07 | 0.01 |
| Tb. Sp, mm |  | 0.397±0.028 | 0.340±0.044 | 0.6 |  | | 0.394±0.038 | | 0.398±0.059 | | 0.8 |  | 0.7 | 0.9 | 1.0 |
| Tb. BV, mm^3^ |  | 0.14±0.02 | 0.17±0.03 ^a^ | 18.8 |  | | 0.16±0.11 | | 0.16±0.04 | | 0.2 |  | 0.6 | 0.7 | 0.9 |
| Tb. TV, mm^3^ |  | 1.76±0.17 | 1.69±0.13 ^a^ | -4.0 |  | | 1.79±0.23 | | 1.67±0.10 ^a^ | | -6.54 |  | < 0.001 | 0.6 | 0.7 |
| Tb. BMD, mg HA/mm^3^ |  | 819.1±31.1 | 835.6±23.8 ^a^ | 2.0 |  | | 813.0±19.9 | | 830.9±39.1 ^a^ | | 2.2 |  | < 0.001 | 0.8 | 0.3 |
| **CORTICAL, 37%** |  |  |  |  |  | |  | |  | |  |  |  |  |  |
| Ct. Ar, mm^2^ |  | 0.755±0.026 | 1.049±0.075 ^a^ | 39.1 |  | | 0.774±0.039 | | 1.058±0.045 ^a^ | | 36.7 |  | < 0.001 | 0.3 | 0.5 |
| Ct. BV, mm^3^ |  | 0.332±0.017 | 0.456±0.035 ^a^ | 37.4 |  | | 0.335±0.020 | | 0.460±0.018 ^a^ | | 37.4 |  | < 0.001 | 0.5 | 0.8 |
| Imax, mm^4^ |  | 0.220±0.021 | 0.279±0.020 ^a^ | 27.1 |  | | 0.216±0.028 | | 0.288±0.012 ^a^ | | 33.2 |  | < 0.001 | 0.7 | 0.5 |
| Imin, mm^4^ |  | 0.068±0.004 | 0.111±0.013 ^a^ | 62.5 |  | | 0.070±0.010 | | 0.115±0.009 ^a^ | | 65.5 |  | < 0.001 | 0.3 | 0.6 |
| Ct. BMD, mg HA/mm^3^ |  | 1091.8±15.5 | 1121.0±15.3 ^a^ | 2.7 |  | | 1094.5±23.6 | | 1111.5±16.2 ^a^ | | 1.5 |  | < 0.001 | 0.6 | 0.3 |
| **CORTICAL, 50%** |  |  |  |  |  | |  | |  | |  |  |  |  |  |
| Ct. Ar, mm^2^ |  | 0.605±0.022 | 0.839±0.051 ^a^ | 38.7 |  | | 0.610±0.034 | | 0.822±0.035 ^a^ | | 34.6 |  | < 0.001 | 0.8 | 0.2 |
| Ct. BV, mm^3^ |  | 0.267±0.014 | 0.365±0.026 ^a^ | 36.6 |  | | 0.266±0.018 | | 0.361±0.019 ^a^ | | 35.9 |  | < 0.001 | 0.8 | 0.7 |
| Imax, mm^4^ |  | 0.075±0.005 | 0.116±0.011 ^a^ | 55.6 |  | | 0.075±0.008 | | 0.116±0.001 ^a^ | | 54.8 |  | < 0.001 | 0.9 | 0.8 |
| Imin, mm^4^ |  | 0.060±0.004 | 0.082±0.007 ^a^ | 35.4 |  | | 0.060±0.010 | | 0.081±0.010 ^a^ | | 35.1 |  | < 0.001 | 0.8 | 0.9 |
| Ct. BMD, mg HA/mm^3^ |  | 1149.2±25.3 | 1157.2±17.3 | 0.7 |  | | 1151.0±32.7 | | 1171.7±13.9 | | 1.8 |  | 0.05 | 0.4 | 0.4 |

**Supplementary Table S12 and S13.**

Data are presented as Mean ± SD (n = 10-12).
Abbreviations are given in Supplementary Table S9.

The effects of genotype and load and their interaction were tested by the linear mixed model with repeated measures followed by pair-wise comparisons with Bonferroni correction.

Specific p-values are shown. Diff: Percent difference (%) between Loaded and Control limbs calculated as 100*(Loaded – Control)/Control.

^a^ *p* < 0.05 for significant loading effect by the linear mixed model with repeated measures (Loaded versus Control).

^b^ *p* < 0.05 for significant genotype effect on the loaded limb by the linear mixed model with repeated measures.

**^§^** *p* < 0.05 for significant loading effect between genotypes by the linear mixed model with repeated measures.

**Supplementary Table S14**. Fold-change of the expressions of genes in lumbar vertebrae (L3-5) for 12wk (young) and 30wk (adult) old male ERβ-dOT and LC mice.

| **Fold-change**  **L3-5** |  | **12wk** | | **p-value (KO vs. LC)** |  | **30wk** | | **p-value (KO vs. LC)** |
| --- | --- | --- | --- | --- | --- | --- | --- | --- |
|  |  | MLC | MKO |  |  | MLC | MKO |  |
| **ERβ (*Esr2*)** |  | 1.0±1.4 | 1.1±1.2 | 0.5 |  | 1.0±1.3 | 1.2±0.8 | 0.3 |
| **ERα (*Esr1*)** |  | 1.0±0.9 | 2.2±1.7 | 0.1 |  | 1.0±1.1 | 1.1±0.8 | 0.5 |
| **AR (*Ar*)** |  | 1.0±0.7 | 1.4±1.6 | 0.9 |  | 1.0±0.6 | 1.9±1.8 | 0.1 |
| **Aromatase (*Cyp19a1*)** |  | 1.0±1.7 | 0.7±0.6 | 0.5 |  | 1.0±1.3 | 2.4±3.8 | 0.1 |
| **Cat K (*Ctsk*)** |  | 1.0±0.8 | 1.2±0.6 | 0.4 |  | 1.0±0.8 | 1.9±1.9 | 0.3 |
| **RANKL (*Tnfsf11*)** |  | 1.0±0.9 | 0.6±0.7 | 0.3 |  | 1.0±2.1 | 0.2 ±0.3 | 0.6 |
| **OPG (*Tnfsf11b*)** |  | 1.0±1.5 | 1.6±0.8 | 0.1 |  | 1.0±1.7 | 0.3±0.2 | 0.5 |
| **RANKL/OPG** |  | 1.0±0.1 | 0.8±0.3 | 0.2 |  | 1.0±0.1 | 1.0±0.1 | 0.9 |
| **Type-I collagen (*Col1a1*)** |  | 1.0±0.7 | 1.1±0.7 | 0.9 |  | 1.0±0.4 | 1.4±1.4 | 0.8 |
| **M-CSF (*Csf1*)** |  | 1.0±1.3 | 0.6±0.3 | 0.9 |  | 1.0±0.6 | 0.5±0.4 | 0.1 |
| **Sclerostin (*Sost*)** |  | 1.0±1.1 | 1.2±0.6 | 0.3 |  | NA | NA |  |

Data are presented as Mean ± SD (n = 4-10).

Total RNA was extracted from the cancellous bone (with marrow) of the lumbar vertebral body (L3-5) in male ERβ-dOT (MKO) and LC (MLC) mice at 12 wks and 30 wks of age. Markers tested in the RT-qPCR are shown with their gene names in parentheses. Fold-change (FC) gene expression for KO (MKO/FKO) relative to LC (MLC/FLC) (average: 1.0) was calculated using the 2^-△△Ct^ method. The significant difference in gene expressions between genotypes was tested at the delta CT level, which was calculated as CT _test gene_ – CT _housekeeping_, by Student’s *t*-test (p < 0.05). The specific p-values are shown in the table. NA, no measurement.

**
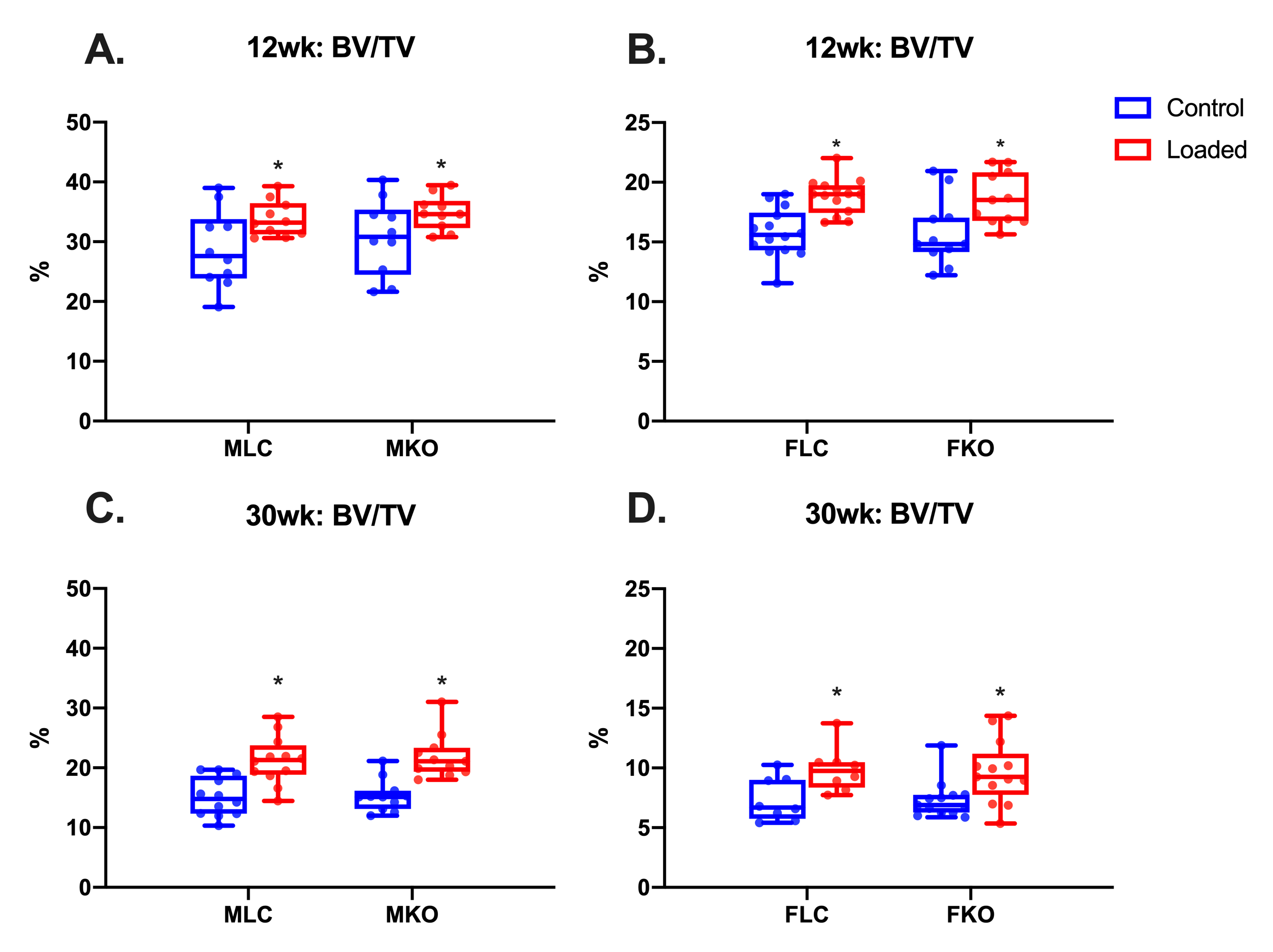
**

**Supplementary Figure S1. Tibial cancellous adaptive response to the compressive loading in 12wk (young) and 30wk (adult) old male and female LC and ERβ-dOT (KO) mice**.

The structural adaptive response in cancellous bone was determined in the loaded (Loaded, red) and controlled (Control, blue) tibiae of male and female LC (MLC, FLC) and ERβ-dOT (MKO, FKO) mice at 12 wks (A, B) and 30 wks of age (C, D) by micro-CT analysis. Trabecular bone volume fraction (BV/TV) of the proximal tibiae is shown.

Data are presented as boxplots with median and interquartile ranges (IQR; 25th to 75th percentile) including all data points (n = 10-12 per group). The effects of genotype and load and their interaction were tested by the linear mixed model with repeated measures followed by pair-wise comparisons with Bonferroni correction. * *p* < 0.05 for significant loading effect for the same genotype by the linear mixed model with repeated measures. Specific p values are shown when there is a significant genotype-load interaction (p < 0.05).
